# Informal coercion during childbirth: risk factors and prevalence estimates from a nationwide survey of women in Switzerland

**DOI:** 10.1186/s12884-021-03826-1

**Published:** 2021-05-10

**Authors:** Stephan Oelhafen, Manuel Trachsel, Settimio Monteverde, Luigi Raio, Eva Cignacco Müller

**Affiliations:** 1 Department of Health Professions, Applied Research & Development in Midwifery, Bern University of Applied Sciences, Murtenstrasse 10, 3008 Bern, Switzerland; 2 Institute of Biomedical Ethics and History of Medicine, University of Zurich, Zurich, Switzerland; 3 Clinical Ethics Unit, University Hospital of Basel and Psychiatric University Clinics Basel, Basel, Switzerland; 4 Department of Health Professions, School of Nursing, Bern University of Applied Sciences, Bern, Switzerland; 5 Department of Obstetrics and Gynecology, University Hospital of Bern, Bern, Switzerland

**Keywords:** Coercion, Informal coercion, Childbirth, Switzerland, Survey, Mode of delivery, Mistreatment, Disrespect, Abuse

## Abstract

**Background:**

In many countries, the increase in facility births is accompanied by a high rate of obstetric interventions. Lower birthrates or elevated risk factors such as women’s higher age at childbirth and an increased need for control and security cannot entirely explain this rise in obstetric interventions. Another possible factor is that women are coerced to agree to interventions, but the prevalence of coercive interventions in Switzerland is unknown.

**Methods:**

In a nationwide cross-sectional online survey, we assessed the prevalence of informal coercion during childbirth, women’s satisfaction with childbirth, and the prevalence of women at risk of postpartum depression. Women aged 18 years or older who had given birth in Switzerland within the previous 12 months were recruited online through Facebook ads or through various offline channels. We used multivariable logistic regression to estimate the risk ratios associated with multiple individual and contextual factors.

**Results:**

In total, 6054 women completed the questionnaire (a dropout rate of 16.2%). An estimated 26.7% of women experienced some form of informal coercion during childbirth. As compared to vaginal delivery, cesarean section (CS) and instrumental vaginal birth were associated with an increased risk of informal coercion (planned CS risk ratio [RR]: 1.52, 95% confidence interval [1.18,1.96]; unplanned CS RR: 1.92 [1.61,2.28]; emergency CS RR: 2.10 [1.71,2.58]; instrumental vaginal birth RR: 2.17 [1.85,2.55]). Additionally, migrant women (RR: 1.45 [1.26,1.66]) and women for whom a self-determined vaginal birth was more important (RR: 1.15 [1.06,1.24]) more often reported informal coercion. Emergency cesarean section (RR: 1.32 [1.08,1.62]), being transferred to hospital (RR: 1.33 [1.11,1.60]), and experiencing informal coercion (RR: 1.35 [1.19,1.54]) were all associated with a higher risk of postpartum depression. Finally, women who had a non-instrumental vaginal birth reported higher satisfaction with childbirth while women who experienced informal coercion reported lower satisfaction.

**Conclusions:**

One in four women experience informal coercion during childbirth, and this experience is associated with a higher risk of postpartum depression and lower satisfaction with childbirth. To prevent traumatic after-effects, health care professionals should make every effort to prevent informal coercion and to ensure sensitive aftercare for all new mothers.

**Supplementary Information:**

The online version contains supplementary material available at 10.1186/s12884-021-03826-1.

## Background

When asked about their preferred way of giving birth, most women favor a vaginal birth with as few interventions as possible, and without anesthesia [1–3]. This preference is not reflected in the high number of obstetric interventions in most middle- and high-income countries [4, 5], even for low-risk pregnancies. For example, large-scale studies in the US and Canada indicate that 60–90% of women who had planned to have a vaginal birth underwent one of the following interventions: induction of labor around term, epidural or spinal anesthesia, amniotomy, episiotomy, instrumental vaginal birth, or cesarean section (CS). Studies in Germany and the US suggest that the observed increase in CS in recent decades is mainly attributable to somewhat subjective criteria or relative indications such as fetal distress or arrest of cervical dilation [6–8]. Other factors such as higher age at childbirth, the related increase in multiple birth rates, or obesity and associated risks cannot entirely explain the increase in CS [9].

Women’s concern to ensure a safe birth may also contribute to the increase in obstetric interventions, although the actual benefits of some interventions are disputed [4, 10]. For example, in a Canadian survey of 6421 women, 79.8% said they were satisfied with their overall birth experience, even though the rate of obstetric interventions was high [11]. In most regions of the world, the average fertility rate has declined by more than 50% over the last hundred years, which may explain the increased emphasis on safety and control during pregnancy and childbirth [7, 12]. Some women actually favor interventions for a quicker and ideally painless childbirth rather than a vaginal birth with no intervention [2], especially if they feel anxious about giving birth or have had previous negative experiences [3, 5].

Given most women’s expressed preference for a vaginal birth and the effect of social conditions on preferences, the extent to which the increase in obstetric interventions reflects their own safety concerns or medical indications remains unclear. Importantly, this also raises questions about the role of informal coercion in seeking women’s consent to interventions. The term *informal coercion* encompasses a range of measures on the continuum between self-determination and formal coercion, including inducement, persuasion, manipulation, pressure, and threats (cf. [13–19]). In most jurisdictions, *formal* coercion during birth is only permissible under specific circumstances—i.e., when women lack decision-making capacity [20]. In psychiatry, informal coercion is sometimes advocated as a means of avoiding formal coercion [21, 22] such as forced medication or feeding. In obstetrics and gynecology, formal coercion is far less common because women in labor generally have decision-making capacity. However, informal coercion might be used during childbirth to urge women to accept obstetric interventions. From an ethical and legal standpoint, such interventions are admissible only if the woman can accept or decline them freely, with proper information and guidance from health care professionals (HCP), and without undue influence or coercion [23].

Research on the quality of maternity care in low-income countries has reported evidence of inadequate professional standards, including disrespectful, abusive, or violent behaviors [2, 24–26]. In high-income countries, subtler approaches such as informal coercion may be more prevalent, as health care systems emphasize respect for patient autonomy and human rights [24]. Vedam et al. [27] reported that many women feel coerced by HCP, and that their physical needs and complaints are trivialized. In a representative study of 2400 women in the US, about 15% of those who underwent induction of labor, epidural anesthesia, or CS felt pressured to accept the treatment, and about half of those who favored a vaginal birth rather than a CS were not afforded this opportunity [1]. For women who felt pressured, the risk of labor induction was twice as high, and the risk of CS was six times as high even in the absence of any medical indication. In another cross-sectional study of 2700 women in the US, 28% of women who gave birth in hospital reported some form of mistreatment—most often in the form of unsupportive care, being shouted at or scolded, violation of privacy, or being forced to accept specified treatments [24].

While conflicts between women and HCP may be quite open, informal coercion can take covert forms that may or may not be apparent to women or HCP. For example, HCP report that they frequently “pull the dead baby card”, holding the mother responsible for a potential adverse outcome, regardless of whether the baby is actually at risk [28, 29]. It follows that women’s reports of coercion depend on their level of knowledge about childbirth in general and about the rationale for a given obstetric intervention [28, 30].

As the extent of restrictions on women’s self-determination during childbirth remains unknown, the goals of the current study were a) to assess the prevalence and forms of informal coercion during childbirth in Switzerland; b) to identify individual and contextual factors that contribute to informal coercion; and c) to determine whether and how informal coercion is associated with childbirth satisfaction and postpartum depression.

## Methods

### Design

The nationwide cross-sectional survey sampled women aged 18 years or older who had given birth in Switzerland within the previous 12 months. The recruitment phase lasted from August 2019 to January 2020. Respondents completed a self-administered online questionnaire.

### Recruitment

Following Vehovar et al. [31], recruitment procedures included both offline and online strategies. Candidates were recruited online through paid Facebook ads that redirected them to the questionnaire on clicking. For offline recruitment, 180 pediatric or gynecological practices were selected randomly from an online phone directory (https://tel.search.ch/), and each practice received 10 leaflets for distribution to women who met the inclusion criteria. In each Swiss canton, the number of selected medical practices reflected the regional proportion of overall births; the smaller French- and Italian-speaking parts of Switzerland were slightly oversampled as compared to the German-speaking part. Through the newsletter of the Swiss Midwives Association, we also asked midwives to distribute a direct survey link to women who met the criteria, and an ad was also posted in two Swiss parenting magazines. Women recruited through the parenting magazines and the leaflets in medical practices had to enter a short vanity URL (e.g., bfh.ch/birthstudy) to access the survey, which was available in four languages: German, French, Italian, and English.

To minimize selection bias, the recruitment material used neutral images and videos and vague wording [32] because ad content impacts Facebook’s delivery algorithms and influences the likelihood of response [33]. In both the leaflets and the Facebook ads, the headline read, “How did you experience the birth of your child?”. For the leaflets, we chose an image of a newborn being held in someone’s hands (AdobeStock #193629437), and the Facebook ad included a short video close-up of a newborn on its mother’s chest (AdobeStock #120284636).

Recruitment on Facebook took place in four waves. Campaigns were stratified either by language region (German-, French-, and Italian-speaking) or by age category; the budgets allocated to each stratum facilitated slight oversampling of smaller strata as defined by the most recent national census data [34]. After three recruitment waves, it became apparent that migrants were underrepresented in some age groups. For that reason, the final wave defined strata in terms of the joint distribution of age categories and residency status, and budgets were increased accordingly for underrepresented groups.

### Questionnaire development

The survey combined items from existing questionnaires and self-developed items. As a first step in questionnaire development, we completed a review of the literature on questionnaires that assess childbirth experience, patient satisfaction, patient participation, autonomy and respect, informal coercion, informed consent, mistreatment and abuse during childbirth [1, 13, 14, 16, 27, 35–40]. Relevant items were then adapted for the present study; in particular, as the goal was to achieve a low dropout rate, we developed a relatively short questionnaire, placing potentially more “interesting” questions about the birth itself at the beginning [41]. To minimize any bias favoring more highly educated women, questions were worded to ensure both medical precision and lay understanding. For example, to assess high-risk pregnancy, we asked women whether they had required in- or outpatient medical treatment during pregnancy rather than presenting a long checklist of complications and diseases.

A first draft of the questionnaire was reviewed by 19 experts (eight obstetricians, nine midwives, one clinical ethicist, and one clinical psychologist), who rated the relevance and clarity of each question on a 4-point Likert scale and commented on issues or suggested changes. The content validity index for single items ranged between 0.82 and 1.00 (*M* = 0.95) [42], and rated intelligibility ranged between 0.76 and 1.00 (*M* = 0.82). A second draft was then piloted with 20 mothers, who completed the questionnaire and commented on the intelligibility of the questions and any missing elements. The questionnaire was then professionally translated into French, Italian, and English, and all items were then reviewed by three lab members who were fluent in the target languages. As a final step, all four versions were compared item by item to ensure consistency across the four languages.

The questionnaire was implemented using Qualtrics™, focusing on mobile phone compatibility. Response options were presented in random order unless there was a natural order (e.g., mother’s age category). When asked about informal coercion, forced-choice questions were used rather than a *select-all-that-apply* option, as these are thought to provide more accurate responses regarding undesirable events [43, 44]. On the assumption that items related to informal coercion might trigger additional thoughts, we included multiple open questions, which is believed to increase response rates [41].

### Outcome variables

Informal coercion was operationalized in line with the Swiss Academy of Medical Sciences’ guidelines on coercive measures in medicine [45] and was assessed at two levels. First, all respondents were asked whether they had felt pressured to consent to any intervention (see *General,* Table 1). Second, women who had undergone CS, instrumental vaginal birth, induction of labor, episiotomy, or amniotomy were asked six questions about the specific intervention, addressing issues of informed consent, opposition to the intervention, and intimidation or manipulation by HCP (see *Intervention-specific*, Table 1). The questionnaire design facilitated assessment of the prevalence of informal coercion (regardless of whether women had undergone obstetric intervention) and more precise evaluation of informal coercion in the context of specific interventions. Additionally, one item (*Insult*) assessed verbal violence: “Did any health professional address you in an insulting or derogatory manner?”

**Table 1:** General and intervention-specific items measuring informal coercion

General	*Pressure*	Did any of the health professionals pressure you into agreeing to an examination or medical intervention?
Intervention-specific	*Information*	I was adequately informed about the pros and cons.
	*Time*	I had enough time to think about it before having to decide.
	*Agreement*	I agreed with the decision.
	*Opposition*	I spoke out against the measure.
	*Intimidation*	I had been made anxious that something could happen to me or my child if I did not agree to the procedure.
	*Manipulation*	I feel like I was given information designed to coax me into agreeing to the procedure.

Only women who had undergone a specified obstetric intervention were asked intervention-specific questions, which addressed only the relevant intervention (e.g., “Which of the following statements apply to the induction of labor?”)

Initially, we considered the criteria for informal coercion to be fulfilled if at least one of the seven responses indicated that the woman had experienced informal coercion. However, we subsequently adopted a more conservative and reliable measure for a number of reasons. First, several conceivable response patterns do not imply coercion. For example, if a woman replied that she felt intimidated but also felt well informed or agreed with the intervention, it would be inappropriate to assume coercion. Similarly, if a woman stated that she was not sufficiently informed (*Information*) and did not have enough time to reflect (*Time*), these two responses alone do not necessarily imply informal coercion, since there might have been justified time pressure regarding an intervention. On that basis, respondents were categorized as having experienced informal coercion during childbirth if they felt pressured to consent to any medical intervention (*Pressure)* and/or if they reported two or more intervention-specific forms of informal coercion (but not *Information* and *Time* alone).

Satisfaction with childbirth was measured using the 12-item short version of Salmon’s item list (SIL) [46], which is a multidimensional instrument covering fulfilment, physical discomfort, and emotional adaptation. The internal consistency of the scale used in the current study (Cronbach’s alpha = 0.88, *n* = 6017; based on all complete SIL responses) was comparable to that reported by Stadlmayr et al. [47] for the full 20-item version (α = 0.87, *n* = 251). While the original instructions state that respondents should reply with regard to the whole birthing process (including “the first hours after birth”), we asked women about the feelings that best described their “childbirth experience.”^1^

To test for a possible association between the experience of informal coercion and postpartum depression, we used the two validated Whooley questions [48]. In their diagnostic meta-analysis, Bosanquet et al. [49] reported a pooled sensitivity of 0.95 and a pooled specificity of 0.65 to identify depression in a general sample using these two questions. Other studies of women in both the pre- and postnatal period, have reported no major differences in diagnostic performance between the Whooley Questions and the 12-item Edinburgh Postnatal Depression Scale [50, 51]. Although the Whooley Questions may lack specificity and indicate any mental health disorder [50], the National Institute for Health and Care Excellence [52] recommends their use to identify depression in the early postnatal period.

### Predictors and possible confounders

Based on our literature review and the empirical findings outlined in the Introduction, we collected data on sociodemographics, birth preparation, birth setting, birth history, pregnancy and birth characteristics, preference for active participation during childbirth, medical indications, medication, freedom of movement, and other obstetric interventions.^2^ All of the study variables are listed in Additional file 1: Tables S1a-d.

### Data analysis

As the primary goal of the statistical analysis was to estimate the prevalence of informal coercion among new mothers in Switzerland, appropriate procedures were applied to weight the survey sample accordingly. A raking algorithm was chosen on the basis of full joint distribution of age category and residence status and marginal distributions of civil status, place of birth, mode of delivery, nulliparity, and geographical region. Any weights greater than five times the average were subsequently trimmed [53–55]. The calculation of weights was based on the most recent available data from the Swiss Federal Statistical Office [34, 56–58]. For non-hospital births, we used data provided by the Swiss interest group of birthing centers (IGGH-CH®) and the Swiss Association of Midwives [59, 60].

All analyses were performed using R 3.6.3 software [61]. Data were first imported after using the qualtRics package [62]. Missing data from the completed questionnaires were imputed using the MICE package [63] and default imputation methods, comprising 50 imputations and a maximum of 30 iterations. Imputed datasets were then weighted using the survey package’s rake procedure [64]. Associated risk ratios (RR) were based on multivariable logistic regression models and on multivariable Poisson regression models for prevalence ≥10% [65]. We included all potentially relevant predictors for informal coercion, satisfaction with childbirth, and postpartum depression. Optimal scaling procedures [66, 67] and subsequent principal component analysis were used to reduce the dimensionality of a few selected variables; those with arbitrary numerical scales were standardized to enhance predictor comparability.

One researcher categorized responses to *Other* options or open-ended questions, and discussed ambiguous cases with a second researcher. Responses to open-ended questions were used to validate responses to closed questions and to correct or delete entries that were clearly erroneous. Comments in *Other* responses were assigned either to existing response options or to a separate category.

## Results

### Survey response

In total, 7663 women accessed the first survey page and provided informed consent. Most participants (6625, 86.5%) were recruited through Facebook; the remainder (1026, 13.5%) were recruited through other channels. Of these, 428 women (5.6%) were excluded from the final analysis because their most recent birth was more than 12 months previously. Additionally, 16 responses (0.22%) were excluded after checking the comments, mainly because the birth was not in Switzerland or because they were duplicate entries. Of the remaining 7226 women who started the questionnaire and met all of the eligibility criteria, 6054 (83.8%) completed it. Regarding missing data in all completed questionnaires, one question (birth duration) had 10.4% missing data, six items had less than 4% and all other items had less than 1%.

### Demographics

Table 2 shows descriptive statistics for selected demographic information and birth characteristics in both the survey and census data. The census data were used to weight the sample data for all subsequent analyses. The survey sample overrepresented Swiss women who had a non-instrumental vaginal delivery and did not give birth in a hospital. Descriptive statistics for additional sociodemographic variables and pregnancy and birth characteristics of the survey sample are summarized in Additional file 2: Table S2.

**Table 2:** Selected demographic and birth-related variables: final survey and census data

	Survey data	Census data
	*(N = 6054)*	*(N = 86,132)*
Maternal age (years)		
18–23	195 (3.2%)	3130 (3.6%)
24–27	707 (11.7%)	10,498 (12.2%)
28–31	1682 (27.8%)	22,940 (26.6%)
32–35	1950 (32.3%)	26,984 (31.3%)
36–39	1179 (19.5%)	16,573 (19.2%)
40+	332 (5.5%)	6007 (7.0%)
Marital status		
Married/Registered partnership	4440 (73.5%)	63,359 (73.6%)
Single*	1601 (26.5%)	22,773 (26.4%)
Nationality		
Swiss	4927 (81.6%)	51,772 (60.1%)
Neighboring state	607 (10.1%)	9810 (11.4%)
Other	504 (8.4%)	24,550 (28.5%)
Major regions		
Espace Mittelland	1666 (27.6%)	18,392 (21.4%)
North-West Switzerland	757 (12.5%)	11,643 (13.5%)
Eastern Switzerland	706 (11.7%)	11,645 (13.5%)
Lake Geneva Region	1203 (19.9%)	17,085 (19.8%)
Central Switzerland	539 (8.9%)	8290 (9.6%)
Ticino	285 (4.7%)	2493 (2.9%)
Zurich	879 (14.6%)	16,584 (19.3%)
Place of birth		
Hospital	5457 (90.6%)	83,256 (96.7%)
Birthing center	338 (5.6%)	2151 (2.5%)
At home	228 (3.8%)	725 (0.8%)
Mode of delivery		
Non-instrumental vaginal birth	3952 (65.3%)	49,429 (57.4%)
Forceps or vacuum birth	693 (11.4%)	9492 (11.0%)
Cesarean section	1409 (23.3%)	27,211 (31.6%)
Nulliparous	3505 (57.9%)	41,734 (48.5%)

### Informed consent and informal coercion

Table 3 shows descriptive data for the three aspects of informed consent (*Information*, *Time*, *Agreement*) and the three forms of informal coercion (*Opposition*, *Intimidation*, *Manipulation*) for different delivery modes and selected interventions. Similar procedures or procedures that co-occur frequently exhibited a similar pattern of ratings for informed consent and informal coercion. Women who had a planned CS reported high levels of being adequately informed, having enough time to decide, and agreeing with the decision. In comparison, women who underwent an unplanned CS or induction of labor (which often co-occur) reported lower levels of *Information* and *Time* and returned the highest ratings for opposing the procedure and feeling manipulated. Overall, emergency CS is associated with the highest rate of informal coercion, as 37% of those who had an emergency CS felt intimidated. The lowest ratings for informed consent relate to instrumental birth and episiotomy; only about 30% of the women felt adequately informed regarding both interventions. Only about 20% of the women who had an instrumental birth and 17% of those who had an episiotomy felt they had sufficient time to make their decision. Finally, only about half of the women who had an amniotomy received adequate information about the procedure (53%) and had enough time to decide (56%).

**Table 3:** Absolute and relative frequencies of informed consent and informal coercion by delivery mode or intervention

	Aspects of informed consent			Forms of informal coercion		
	Information	Time	Agreement	Opposition	Intimidation	Manipulation
Planned cesarean section	427 (90.7%)	427 (90.7%)	451 (95.8%)	35 (7.4%)	142 (30.1%)	25 (5.3%)
Unplanned cesarean section	384 (76.2%)	322 (63.9%)	481 (95.4%)	43 (8.5%)	111 (22.0%)	36 (7.1%)
Emergency cesarean section	199 (58.5%)	98 (28.8%)	311 (91.5%)	20 (5.9%)	126 (37.1%)	13 (3.8%)
Forceps or vacuum birth	213 (30.7%)	143 (20.6%)	616 (88.9%)	22 (3.2%)	114 (16.5%)	27 (3.9%)
Induction of labor	1136 (74.2%)	1122 (73.3%)	1384 (90.5%)	152 (9.9%)	407 (26.6%)	151 (9.9%)
Amniotomy	713 (52.5%)	754 (55.5%)	1277 (94.0%)	32 (2.4%)	59 (4.3%)	50 (3.7%)
Episiotomy	208 (30.0%)	119 (17.2%)	530 (76.5%)	45 (6.5%)	89 (12.8%)	38 (5.5%)

Pairwise associations between informal coercion and medical indications as reported by the women can be found in Additional file 3: Table S3. Overall, women reported higher levels of informal coercion when they did not understand the reason for the intervention. All other interventions (e.g., fundal pressure, vaginal examinations, medication) were associated with a higher risk of informal coercion. In contrast, the risk was lower for women who had the opportunity to discuss the birth afterwards with the HCP involved.

Using imputed and weighted data, the estimated probability of experiencing any form of informal coercion was 26.7%. Furthermore, 16.3% of women reported pressure to consent and 9.5% reported being treated in a derogatory or insulting manner at least once. Risk ratios and 95% confidence intervals (CI) for factors associated with informal coercion are shown in Table 4 (left column). Women from a non-neighboring state were at greater risk of informal coercion (RR 1.45, 95% CI [1.26,1.66]), as were women living in more urban cantons (RR 1.16 [1.09,1.23]). A preference for autonomy in decision-making during childbirth (RR 1.15 [1.10,1.21]) and for vaginal birth (RR 1.15 [1.06,1.24]) increased the risk of informal coercion, as did high-risk pregnancy (RR 1.25 [1.10,1.41]). In contrast, for women who gave birth at a birthing center (an independent birth facility run by midwives), the risk was three times lower (RR 0.35 [0.21,0.59]). Women who did not give birth where they had initially planned because they had to be transferred from a birthing center or a different hospital were also at greater risk of informal coercion (RR 1.47 [1.25,1.73]). In addition, instrumental vaginal birth and all types of CS were associated with a higher risk of informal coercion (all RRs > 1.5). Interestingly, women reported informal coercion more often where more time had elapsed since the birth (RR 1.17 [1.06,1.29]).

**Table 4:** Estimated risks associated with informal coercion, postpartum depression, and satisfaction with childbirth

	Risk ratios				*β* estimate	
	Informal coercion		Postpartum depression		Satisfaction with childbirth	
	RR	95% CI	RR	95% CI	*β*	95% CI
**MATERNAL SOCIODEMOGRAPHIC CHARACTERISTICS**						
Maternal age (years):						
18–23	Ref.		Ref.		Ref.	
24–27	1.06	[0.77,1.47]	1.25	[0.91,1.72]	1.47	[−1.29,4.22]
28–31	1.03	[0.76,1.41]	1.10	[0.80,1.51]	1.71	[−0.90,4.32]
32–35	0.88	[0.63,1.22]	1.03	[0.75,1.43]	0.30	[−2.35,2.95]
36–39	0.91	[0.65,1.28]	0.97	[0.69,1.36]	1.06	[−1.71,3.82]
40+	0.94	[0.64,1.39]	0.95	[0.64,1.43]	1.67	[−1.52,4.86]
Nationality:						
Swiss	Ref.		Ref.		Ref.	
Neighboring state	1.06	[0.91,1.25]	1.50	[1.29,1.74]	−0.99	[−2.24,0.26]
Other	1.45	[1.26,1.66]	1.57	[1.36,1.82]	0.01	[−1.22,1.24]
Socioeconomic status (+ 1 SD)	1.02	[0.96,1.09]	0.90	[0.85,0.96]	−0.02	[−0.52,0.49]
Urban (+ 1 SD)	1.16	[1.09,1.23]	1.07	[1.00,1.14]	−0.24	[− 0.64,0.16]
**MOTHERS’ PREFERENCE AND EXPECTATIONS**						
Preference for autonomous decision (+ 1 SD)	1.15	[1.10,1.21]	1.02	[0.96,1.08]	0.37	[−0.08,0.82]
Preference for vaginal birth (+ 1 SD)	1.15	[1.06,1.24]	1.02	[0.95,1.09]	−0.74	[−1.25,-0.23]
Birth preparation (+ 1 SD)	1.06	[1.00,1.13]	0.98	[0.92,1.05]	0.16	[−0.31,0.63]
**PREGNANCY CHARACTERISTICS**						
Parity:						
Nulliparous	Ref.		Ref.		Ref.	
Multiparous - no previous CS	0.94	[0.79,1.13]	0.90	[0.76,1.06]	1.35	[0.21,2.49]
Multiparous - previous CS	1.00	[0.81,1.23]	0.92	[0.74,1.14]	1.50	[−0.04,3.05]
Multiple birth	1.68	[1.20,2.35]	0.87	[0.59,1.29]	1.27	[−2.03,4.56]
High-risk pregnancy	1.25	[1.10,1.41]	1.04	[0.92,1.19]	−1.76	[−2.73,-0.79]
Main caregiver:						
Physician	Ref.		Ref.		Ref.	
Midwife	1.11	[0.93,1.32]	0.88	[0.73,1.07]	−0.17	[−1.49,1.16]
Both	0.95	[0.82,1.10]	0.82	[0.69,0.97]	0.99	[−0.08,2.06]
Other	1.58	[0.77,3.27]	0.89	[0.37,2.11]	−6.12	[−13.55,1.3]
**BIRTH SETTING**						
Knew at least one of the care providers	1.00	[0.88,1.14]	0.98	[0.86,1.12]	1.05	[0.09,2.00]
Place of birth:						
Public hospital	Ref.		Ref.		Ref.	
Private hospital	1.01	[0.86,1.19]	1.05	[0.89,1.24]	0.19	[−0.95,1.34]
Birthing center	0.35	[0.21,0.59]	0.65	[0.46,0.93]	3.54	[1.96,5.13]
At home	0.72	[0.44,1.20]	0.88	[0.56,1.37]	7.55	[5.84,9.27]
Unplanned place of birth	1.47	[1.25,1.73]	1.33	[1.11,1.60]	−3.25	[−5.19,-1.31]
**BIRTH CHARACTERISTICS**						
Mode of birth:						
Non-instrumental vaginal birth	Ref.		Ref.		Ref.	
Forceps or vacuum birth	2.17	[1.85,2.55]	1.00	[0.84,1.20]	−6.94	[−8.41,-5.48]
Planned cesarean section	1.52	[1.18,1.96]	1.00	[0.79,1.26]	−2.31	[−4.08,-0.54]
Unplanned cesarean section	1.92	[1.61,2.28]	0.90	[0.72,1.13]	−9.35	[−11.14,-7.56]
Emergency cesarean section	2.10	[1.71,2.58]	1.32	[1.08,1.62]	− 12.12	[− 14.28,-9.96]
Duration of birth (+ 10 h)	1.07	[1.01,1.13]	1.02	[0.96,1.09]	−2.68	[−3.27,-2.09]
Child’s weight (+ 1000 g)	1.10	[0.96,1.26]	1.02	[0.90,1.16]	0.23	[−0.74,1.20]
Child’s age (+ 6 Mt.)	1.17	[1.06,1.29]	0.89	[0.81,0.99]	0.09	[−0.61,0.79]
**EXPERIENCE OF INFORMAL COERCION**	–	–	1.35	[1.19,1.54]	−7.52	[−8.63,-6.41]

Risk ratios (RR) reflect the risk of informal coercion and postpartum depression. RRs were estimated using multivariable logistic regression and based on an imputed and weighted dataset. *β* coefficients reflect the change of a predictor in the total SIL score as estimated using multivariable linear regression and based on an imputed and weighted dataset. The 95% confidence intervals (CI) are shown in square brackets. All models are controlled for mother’s civil status, health insurance (general, semi−/private), gestational age, and recruitment channel (i.e., Facebook or other). Models for postpartum depression and satisfaction with childbirth are additionally controlled for neonatal intensive care unit transfer (not shown in table)

### Postpartum depression

Responses to the Whooley questions used for depression screening indicated that 27.0% of the women were at risk of postpartum depression or another mental health disorder. Several demographic and birth-related factors were associated with increased risk of possible mental health problems. Women living in urban cantons (RR 1.07 [1.00,1.14]) and migrant women were at greater risk (both RRs > 1.5). Women who gave birth at a birthing center were at lower risk (RR 0.65 [0.46,0.93]), but women who were transferred to a (different) hospital were at higher risk (RR 1.33 [1.11,1.60]). Of all modes of delivery, only emergency CS was associated with increased risk (RR 1.32 [1.08,1.62]). Experiencing informal coercion also increased the risk of postpartum mental health disorders (RR 1.35 [1.19,1.54]).

### Satisfaction

Satisfaction with childbirth was measured as total SIL score; higher values indicated higher satisfaction. The main factors influencing satisfaction were informal coercion, place of birth, and mode of delivery. Experiencing informal coercion had a negative effect on reported childbirth experience (− 7.52 [− 8.63,-6.41]). Women who gave birth at home or at a birthing center were generally more satisfied than women who gave birth at a hospital (birthing center + 3.54 [1.96,5.13]; at home + 7.55 [5.84,9.27]). Women who did not give birth where they had planned to were less satisfied (− 3.25 [− 5.19,-1.31]). Finally, women who had an unplanned or an emergency CS returned the lowest satisfaction ratings (unplanned CS -9.35 [− 11.14,-7.56]; emergency CS -12.12 [− 14.28,-9.96]).

## Discussion

The goals of the current study were to estimate the prevalence of informal coercion during childbirth in Switzerland and to assess the risk associated with a number of individual and contextual factors. To that end, we developed a comprehensive questionnaire addressing various aspects of informal coercion, satisfaction with childbirth, and postpartum depression, as well as a range of demographic, pregnancy, and birth-related characteristics. Women aged 18 years or older who had given birth in Switzerland within the previous 12 months were recruited through online and offline channels; a majority accessed the questionnaire by clicking on a Facebook ad. An estimated 27% of women experienced informal coercion during childbirth, and about 16% reported feeling pressured to consent to an intervention. In addition, the present data demonstrate that informal coercion negatively affects satisfaction with childbirth and is associated with increased risk of postpartum depression.

The observed association between informal coercion and depression does not support conclusions regarding any causal relationship. While experiencing informal coercion may increase the risk of postpartum depression, another possibility is that women who are already suffering from depression may be more likely to experience informal coercion. Nevertheless, these results highlight the importance of safeguarding against informal coercion to prevent post-partum depression. Longitudinal studies have reported that CS may negatively affect women’s delivery experience and increase their subsequent risk of postpartum depression, especially among those with a strong preference for vaginal delivery [68, 69]. Our data suggest that the relationship between mode of delivery and postpartum depression may be mediated by informal coercion, and this seems a worthwhile avenue for future research.

Although their scope and methodology differ, other studies report similar rates of informal coercion in high-income countries. For instance, Vedam et al. [24] found that about 28% of women who gave birth at a hospital in the US experienced mistreatment—most commonly, violations of physical privacy, being shouted at or scolded, and requests for help that were unanswered. In another US study, about 15% of women reported feeling pressured to consent to a medical intervention [1]. In general, the risk of experiencing mistreatment during childbirth seems to be lower for women who are multiparous, older than 30, white, and speak the same language as the HCP. That risk is higher if women have to be transferred to hospital from a different location during childbirth [24, 70]. In line with previous research, the present study indicates that migrant women are at greater risk of experiencing informal coercion than Swiss nationals. The risk is also higher for women living in more urban regions than for those living in more rural regions. In urban regions, the known higher rate of CS [71] and the increased risk of informal coercion identified here suggest that many interventions in urban areas are performed without explicit consent, but this requires further exploration.

Another important unresolved question is how the relationship between birth setting and a woman’s childbirth preferences and expectations impacts the experience of informal coercion. Our data show that women who express a strong preference for a vaginal and self-determined birth tend to report informal coercion more often than women for whom these issues are less important. Expectations related to self-determined birth may reflect different conceptions of a “good” birth [72] that are not always realistic. In institutional settings, women and HCP share the responsibility for the health of both mother and child. Additionally, the birth process in institutional settings is likely to be standardized for reasons of quality and effectiveness, and HCP are required to follow specific guidelines. At first glance, birthing centers may seem to allow for greater self-determination, but any direct comparison between hospitals and birthing centers must be drawn with caution for a number of reasons. First, only women with low-risk pregnancies can give birth at a birthing center. Second, birthing centers are not authorized to carry out the most debated obstetric interventions that incur a higher risk of informal coercion. Finally, the observed association between informal coercion and transfer to hospital does not necessarily imply any misconduct by HCP at the hospital; it might equally reflect a woman’s disappointment—even if she understands the reasons—that her preference for a vaginal birth with minimal medical intervention could not be met. In short, while women’s preferences and expectations clearly impact the experience of informal coercion, the role of the birth setting and associated preferences and expectations require further investigation.

In addition, informal coercion does not necessarily imply HCP intent. While organizational or working conditions can never justify the use of informal coercion, a number of circumstances may explain why it nevertheless occurs (and suggest how it might be prevented). First, economic pressures, incentive systems, or fear of legal liability may cause HCP to feel compelled to intervene when in doubt [18]. Second, most of the interventions performed by HCP are routine operations, and HCP may not always be sensitive to the possible consequences for the mother. Third, women differ in their perception of such suggestions as “support”, “nudge” [73, 74], or outright pressure, and that perception may also change over time [75]. Finally, HCP encounter a wide range of patient attitudes, preferences, and needs, and some may not know how best to respond if a woman rejects a treatment suggestion, even (or especially) if it is based on current best practice [29]. Nevertheless, our findings indicate that informal coercion is a common feature of childbirth, with potentially traumatizing consequences that are likely to affect both the woman and her family. In general, as obstetric interventions seem to have a negative impact on women’s birth experience [56], HCP must take account of potentially harmful outcomes that include both the immediate physical circumstances and longer-term psychological consequences for the mother when contemplating any such intervention.

In the present study, about one in four women reported feeling intimidated during childbirth. This number increased to one in three among women who underwent a CS or induction of labor. While many women favor vaginal birth, concerns about the child’s health tend to overrule other arguments when discussing possible interventions [76]. This explains why “playing the dead baby card” is so effective, as none of those involved—mother or HCP—want to be responsible for a negative outcome [77]. HCP have the power to modulate women’s fears, either frightening them by stressing the possible risks or empowering them to play an active role in childbirth. Clearly, some women also hold false beliefs because of their lack of knowledge about certain interventions. In the present case, women who did not understand the reasons for an intervention were more likely to feel coerced. Women who were afforded the opportunity of a childbirth debriefing to discuss the birth with the HCP involved reported lower rates of informal coercion than those who had no such opportunity. These findings highlight the need for HCP to explain any intervention and the reasons for it [1, 76]. Informing women about procedures and seeking their active participation is not only a legal requirement but a sign of respect for the mother and her child.

### Strengths and limitations

To our knowledge, this is the first in-depth investigation of the prevalence of informal coercion in a large nationwide sample. As well as controlling for multiple characteristics of pregnancy and birth, we controlled for birth preparation and attitudes and expectations regarding patient involvement. The questionnaire design ensured a relatively low dropout rate, yielding a more representative sample.

One significant limitation of the study is the possibility of self-selection bias, which is typically more problematic in non-probability samples. The survey sample was not representative in terms of variables like place of birth, nationality, mode of delivery, and other potentially relevant characteristics that were not assessed. For example, the proportion of women who gave birth at a birthing center was higher than in the census data, which may indicate that the participants were more actively engaged with the topic of childbirth and therefore more interested in responding to the survey. In addition, while the recruitment material was carefully selected, we had no control over or insight into Facebook’s algorithms for delivering ads [33]. On the other hand, we did follow recommended practice to reduce bias in non-probability samples by combining various offline and online recruitment channels [31]. Women recruited through Facebook did not differ significantly from women recruited through other channels in terms of reported informal coercion, postpartum depression, or satisfaction with childbirth.

It is reasonable to assume that the reported prevalence is a fairly conservative estimate, as more satisfied patients are generally more inclined to respond to questionnaires measuring patient satisfaction [78]. In the present case, for example, women were more likely to drop out of the study if they had had an unwanted CS than if the CS was their own preference. Although we used multiple items to assess informal coercion, the rate of covert coercion (i.e., coercion that the women themselves did not recognize) remains completely unknown. These micro-interactions are subtle, and women who are overwhelmed and focused on the birth itself may be unaware of what is going on around them [79]. These feelings may continue for several months, which would explain why women were less likely to report informal coercion in the first few months after birth than around 6 months to a year after.

## Conclusions

More than a quarter of the women who completed our survey reported informal coercion during childbirth—in other words, obstetric interventions that they did not agree with or felt pressured or intimidated to consent to. While all such interventions bore the risk of informal coercion, that risk was higher in cases of induced labor, unplanned CS, emergency CS, or instrumental vaginal birth. As experiences of informal coercion are associated with lower childbirth satisfaction and a higher risk of postpartum depression, it is crucial to make every effort to guard against informal coercion. An increased focus on sensitive aftercare for all new mothers would allow HCP to detect women who have experienced informal coercion and to take the necessary measures to prevent traumatic after-effects. To improve the experience of childbirth, a well-informed and compassionate debate is necessary that must include obstetric interventions and their consequences.

## Supplementary Information

**Additional file 1: Table S1a-d.** List of independent variables, item wording (in English, German, French, and Italian), sources and possible transformations.

**Additional file 2: Table S2.** Descriptive statistics: additional sociodemographic variables, pregnancy, and birth characteristics of the survey sample.

**Additional file 3: Table S3.** Pairwise associations between informal coercion and medical indications, interventions, and diagnostic procedures.

## Data Availability

The individual datasets generated during the present study are not publicly available, as the wide range of variables might deanonymize individual respondents. However, the final dataset used for the analyses is available from the corresponding author on reasonable request.

## Abbreviations

CI: Confidence interval
CS: Cesarean section
HCP: Healthcare professionals
RR: Risk ratio
SIL: Salmon’s item list

## Footnotes

1 Because of a programming error, initially, SIL items were not randomized; this was corrected after 29% of responses had been collected. An additional multivariable linear regression incorporating the factor *SIL randomized yes/no* revealed no significant differences in predictor estimates.

2 Because of a wording error in the survey, the frequencies for gestational age categories are likely to be imprecise. The original *gestational age* item defined the categories (in weeks) as < 32, 32–36, 37–41, and > 42. As this made it impossible to correctly enter a gestational age of 42 weeks, the last category was adjusted to > 41 after 65% of the data had been collected. However, this still leaves room for interpretation. While gestational age did not significantly affect any of the outcomes, we cannot exclude possible effects of this error.

## References

[1] Declercq ER, Sakala C, Corry MP, Applebaum S, Herrlich A (2013). Listening to Mothers^SM^ III: pregnancy and birth.

[2] Downe S, Finlayson K, Oladapo O, Bonet M, Gülmezoglu AM (2018). What matters to women during childbirth: a systematic qualitative review. PLoS One.

[3] Kringeland T, Daltveit AK, Møller A (2010). What characterizes women who want to give birth as naturally as possible without painkillers or intervention?. Sex Reprod Healthc.

[4] Miller S, Abalos E, Chamillard M, Ciapponi A, Colaci D, Comandé D, Diaz V, Geller S, Hanson C, Langer A, Manuelli V, Millar K, Morhason-Bello I, Castro CP, Pileggi VN, Robinson N, Skaer M, Souza JP, Vogel JP, Althabe F (2016). Beyond too little, too late and too much, too soon: a pathway towards evidence-based, respectful maternity care worldwide. Lancet.

[5] Boerma T, Ronsmans C, Melesse DY, Barros AJD, Barros FC, Juan L, Moller AB, Say L, Hosseinpoor AR, Yi M, de Lyra Rabello Neto D, Temmerman M (2018). Global epidemiology of use of and disparities in caesarean sections. Lancet.

[6] Barber EL, Lundsberg LS, Belanger K, Pettker CM, Funai EF, Illuzzi JL (2011). Indications contributing to the increasing cesarean delivery rate. Obstet Gynecol.

[7] Bockenheimer-Lucius G (2002). Zwischen “natürlicher Geburt” und “Wunschsectio”– Zum Problem der Selbstbestimmtheit in der Geburtshilfe. Ethik Med.

[8] Metz TD, Stoddard GJ, Henry E, Jackson M, Holmgren C, Esplin S (2013). How do good candidates for trial of labor after cesarean (TOLAC) who undergo elective repeat cesarean differ from those who choose TOLAC?. Am J Obstet Gynecol.

[9] The Lancet (2018). Stemming the global caesarean section epidemic. Lancet.

[10] Rooks JP (1999). Evidence-based practice and its application to childbirth care for low-risk women. J Nurse Midwifery.

[11] Chalmers B, Dzakpasu S, Heaman M, Kaczorowski J (2008). The Canadian maternity experiences survey: an overview of findings. J Obstet Gynaecol Can.

[12] Uphoff R (2011). Aufklärung und Indikation zur Sectio als Beispiel für geburtshilflichen Paternalismus versus Geburtsmedizin als Dienstleistung für autonome Gebärende. 25 Jahre Arbeitsgemeinschaft - 25 Jahre Arzthaftung.

[13] Szmukler G, Appelbaum PS (2008). Treatment pressures, leverage, coercion, and compulsion in mental health care. J Ment Health.

[14] Schweizerische Akademie der Medizinischen Wissenschaften (SAMW) (2015). Zwangsmassnahmen in der Medizin. Medizinisch-ethische Richtlinien der SAMW.

[15] Dondorp W, de Wert G. Prenatal child protection. ethics of pressure and coercion in prenatal care for addicted pregnant women. In: Hens K, Cutas D, Horstkötter D, editors. Parental Responsibility in the Context of Neuroscience and Genetics: Springer; 2017. p. 121–31. 10.1007/978-3-319-42834-5_8.

[16] Jäger M (2017). Informeller Zwang in der therapeutischen Beziehung. Praxis.

[17] Valenti E, Banks C, Calcedo-Barba A, Bensimon CM, Hoffmann K-M, Pelto-Piri V, Jurin T, Mendoza OM, Mundt AP, Rugkåsa J, Tubini J, Priebe S (2015). Informal coercion in psychiatry: a focus group study of attitudes and experiences of mental health professionals in ten countries. Soc Psychiatry Psychiatr Epidemiol.

[18] Kukura E (2018). Obstetric Violence. Georgetown Law J.

[19] Lorem GF, Hem MH, Molewijk B (2015). Good coercion: patients’ moral evaluation of coercion in mental health care. Int J Ment Health Nurs.

[20] Glezer A (2018). The ethics of court-mandated cesarean sections. J Am Acad Psychiatry Law Online.

[21] Pelto-Piri V, Kjellin L, Hylén U, Valenti E, Priebe S (2019). Different forms of informal coercion in psychiatry: a qualitative study. BMC Res Notes.

[22] Elmer T, Rabenschlag F, Schori D, Zuaboni G, Kozel B, Jaeger S, Mahlke C, Heumann K, Theodoridou A, Jaeger M (2018). Informal coercion as a neglected form of communication in psychiatric settings in Germany and Switzerland. Psychiatry Res.

[23] von Tigerstrom B (2001). Informed consent for treatment: a review of the legal requirements. J Obstet Gynaecol Can.

[24] Vedam S, Stoll K, Taiwo TK, Rubashkin N, Cheyney M, Strauss N (2019). The giving voice to mothers study: inequity and mistreatment during pregnancy and childbirth in the United States. Reprod Health.

[25] Bohren MA, Vogel JP, Hunter EC, Lutsiv O, Makh SK, Souza JP, Aguiar C, Saraiva Coneglian F, Diniz ALA, Tunçalp Ö, Javadi D, Oladapo OT, Khosla R, Hindin MJ, Gülmezoglu AM (2015). The mistreatment of women during childbirth in health facilities globally: a mixed-methods systematic review. PLoS Med.

[26] Bowser D, Hill K (2010). Exploring evidence for disrespect and abuse in facility-based childbirth.

[27] Vedam S, Stoll K, Rubashkin N, Martin K, Miller-Vedam Z, Hayes-Klein H, Jolicoeur G, CCinBC Steering Council (2017). The mothers on respect (MOR) index: measuring quality, safety, and human rights in childbirth. SSM Popul Health.

[28] Hall WA, Tomkinson J, Klein MC (2011). Canadian care providers’ and pregnant Women’s approaches to managing birth: minimizing risk while maximizing integrity. Qual Health Res.

[29] Dexter SC, Windsor S, Watkinson SJ (2013). Meeting the challenge of maternal choice in mode of delivery with vaginal birth after caesarean section: a medical, legal and ethical commentary. BJOG.

[30] Sen G, Reddy B, Iyer A (2018). Beyond measurement: the drivers of disrespect and abuse in obstetric care. Reprod Health Matters.

[31] Vehovar V, Toepoel V, Steinmetz S, Wolf C, Joye D, Smith TW (2016). Non-probability sampling. The Sage handbook of survey methods.

[32] Zhang B, Mildenberger M, Howe PD, Marlon J, Rosenthal SA, Leiserowitz A (2018). Quota sampling using Facebook advertisements. Polit Sci Res Methods.

[33] Ali M, Sapiezynski P, Bogen M, Korolova A, Mislove A, Rieke A (2019). Discrimination through optimization: How Facebook’s ad delivery can lead to skewed outcomes.

[34] Federal Statistical Office (2020). Lebendgeburten nach Alter der Mutter und Kanton, 1970–2019 [Live births by age of mother and canton, 1970–2019].

[35] Vedam S, Stoll K, Martin K, Rubashkin N, Partridge S, Thordarson D, Jolicoeur G, the Changing Childbirth in BC Steering Council (2017). The Mother’s autonomy in decision making (MADM) scale: patient-led development and psychometric testing of a new instrument to evaluate experience of maternity care. PLoS One.

[36] Scholl I, Kriston L, Härter M (2011). PEF-FB-9–Fragebogen zur Partizipativen Entscheidungsfindung (revidierte 9-Item-Fassung). Klinische Diagnostik und Evaluation.

[37] Birthrights (2013). Dignity in Childbirth: the Dignity Survey 2013: Women’s and midwives’ experiences of dignity in UK maternity care.

[38] Schrittenloher V (2015). Peripartale Einflussgrößen auf Geburtmodus und Zufriedenheit unter besonderer Beachtung des Wunschkaiserschnittes: lmu.

[39] Sjetne IS, Bjertnaes OA, Olsen RV, Iversen HH, Bukholm G (2011). The generic short patient experiences questionnaire (GS-PEQ): identification of core items from a survey in Norway. BMC Health Serv Res.

[40] Dencker A, Taft C, Bergqvist L, Lilja H, Berg M (2010). Childbirth experience questionnaire (CEQ): development and evaluation of a multidimensional instrument. BMC Pregnancy Childbirth.

[41] Burns KEA, Duffett M, Kho ME, Meade MO, Adhikari NKJ, Sinuff T, Cook DJ, for the ACCADEMY Group (2008). A guide for the design and conduct of self-administered surveys of clinicians. Can Med Assoc J.

[42] Polit DF, Beck CT (2006). The content validity index: are you sure you know What’s being reported? Critique and recommendations. Res Nurs Health.

[43] Smyth JD, Christian LM, Dillman DA (2008). Does “yes or no” on the telephone mean the same as “check-all-that-apply” on the web?. Public Opin Quart.

[44] Lau A, Kennedy C (2019). When online survey respondents only ‘select some that apply’.

[45] Swiss Academy of Medical Sciences (SAMS) (2015). Medical-ethical guidelines: Coercive measures in medicine.

[46] Stadlmayr W, Bitzer J, Hösli I, Amsler F, Leupold J, Schwendke-Kliem A, Simoni H, Bürgin D (2009). Birth as a multidimensional experience: comparison of the English- and German-language versions of Salmon's item list. J Psychosom Obstet Gynecol.

[47] Stadlmayr W, Amsler F, Lemola S, Stein S, Alt M, Bürgin D, Surbek D, Bitzer J (2006). Memory of childbirth in the second year: the long-term effect of a negative birth experience and its modulation by the perceived intranatal relationship with caregivers. J Psychosom Obstet Gynecol.

[48] Whooley MA, Avins AL, Miranda J, Browner WS (1997). Case-finding instruments for depression. Two questions are as good as many. J Gen Intern Med.

[49] Bosanquet K, Bailey D, Gilbody S, Harden M, Manea L, Nutbrown S, McMillan D (2015). Diagnostic accuracy of the Whooley questions for the identification of depression: a diagnostic meta-analysis. BMJ Open.

[50] Howard LM, Ryan EG, Trevillion K, Anderson F, Bick D, Bye A, Byford S, O'Connor S, Sands P, Demilew J, Milgrom J, Pickles A (2018). Accuracy of the Whooley questions and the Edinburgh postnatal depression scale in identifying depression and other mental disorders in early pregnancy. Brit J Psychiatry.

[51] Shibata Y, Suzuki S (2020). Comparison of the Edinburgh postnatal depression scale and the Whooley questions in screening for postpartum depression in Japan. J Matern Fetal Neonatal Med.

[52] National Institute for Health and Care Excellence (2014). Antenatal postnatal mental health: Clinical management and service guidance (NICE Clinical guideline No. 192).

[53] Kalton G, Flores-Cervantes I (2003). Weighting methods. J Off Stat.

[54] Haziza D, Beaumont J-F (2017). Construction of weights in surveys: a review. Stat Sci.

[55] Lavrakas P (2011). Encyclopedia of survey research methods.

[56] Federal Statistical Office (2020). Lebendgeburten nach Staatsangehörigkeit und Alter der Mutter, 2000–2019 [Live births by nationality and age of mother, 2000–2019].

[57] Federal Statistical Office (2020). Lebendgeburten nach Alter der Mutter und Geburtenfolge, 2005–2019 [Live births by age of mother and birth order, 2005–2019].

[58] Federal Statistical Office (2020). Lebendgeburten nach Geburtenfolge und Zivilstand der Mutter, 2005–2019 [Live births by birth order and marital status of mother, 2005–2019].

[59] Swiss interest group of birthing centers (IGGH-CH®) (2020). Statistik [Statistics].

[60] Swiss Association of Midwives (2019). Statistikbericht der frei praktizierenden Hebammen der Schweiz [Statistical report of the freelance midwives in Switzerland].

[61] R Core Team (2020). R: a language and environment for statistical computing.

[62] Ginn J, Silge J (2020). qualtRics: Download ‘Qualtrics’ Survey Data. R package version 3.1.2.

[63] Buuren SV, Groothuis-Oudshoorn K. mice: Multivariate imputation by chained equations in R. J Stat Software. 2010:1–68. 10.18637/jss.v045.i03.

[64] Lumley T (2019). Analysis of complex survey samples. R package version 3.33–2.

[65] Barros AJ, Hirakata VN (2003). Alternatives for logistic regression in cross-sectional studies: an empirical comparison of models that directly estimate the prevalence ratio. BMC Med Res Methodol.

[66] Mair P, de Leeuw J (2018). Aspect: A General Framework for Multivariate Analysis with Optimal Scaling. R package version 1.0–5.

[67] Nguyen LH, Holmes S (2019). Ten quick tips for effective dimensionality reduction. PLoS Comput Biol.

[68] Eckerdal P, Georgakis MK, Kollia N, Wikström A-K, Högberg U, Skalkidou A (2018). Delineating the association between mode of delivery and postpartum depression symptoms: a longitudinal study. Acta Obstet Gynecol Scand.

[69] Houston KA, Kaimal AJ, Nakagawa S, Gregorich SE, Yee LM, Kuppermann M (2015). Mode of delivery and postpartum depression: the role of patient preferences. Am J Obstet Gynecol.

[70] Kukura E (2009). Choice in birth: preserving access to VBAC. Penn State Law Rev.

[71] Federal Statistical Office (2019). Anzahl und Rate der Kaiserschnitte nach Kanton und Wohnregion [Number and rate of cesarean sections by canton and region].

[72] Karlström A, Nystedt A, Hildingsson I (2015). The meaning of a very positive birth experience: focus groups discussions with women. BMC Pregnancy Childbirth.

[73] Gorin M, Joffe S, Dickert N, Halpern S (2017). Justifying clinical nudges. Hastings Cent Rep.

[74] Lantos JD (2018). Ethical problems in decision making in the neonatal ICU. N Engl J Med.

[75] Soltani H, Dickinson FM, Kalk J, Payne K (2008). Breast feeding practices and views among diabetic women: a retrospective cohort study. Midwifery.

[76] Coates R, Cupples G, Scamell A, McCourt C (2018). Women’s experiences of induction of labour: qualitative systematic review and thematic synthesis. Midwifery.

[77] Oelhafen S, Monteverde S, Cignacco E (2018). Exploring moral problems and moral competences in midwifery: a qualitative study. Nurs Ethics.

[78] French K (1981). Methodological considerations in hospital patient opinion surveys. Int J Nurs Stud.

[79] Darilek U (2018). A Woman's right to dignified, respectful healthcare during childbirth: a review of the literature on obstetric mistreatment. Issues Mental Health Nurs.

