# Correction to: Informal coercion during childbirth: risk factors and prevalence estimates from a nationwide survey of women in Switzerland

**DOI:** 10.1186/s12884-021-03939-7

**Published:** 2021-06-22

**Authors:** Stephan Oelhafen, Manuel Trachsel, Settimio Monteverde, Luigi Raio, Eva Cignacco

**Affiliations:** 1 Department of Health Professions, Applied Research & Development in Midwifery, Bern University of Applied Sciences, Murtenstrasse 10, 3008 Bern, Switzerland; 2 Institute of Biomedical Ethics and History of Medicine, University of Zurich, Zurich, Switzerland; 3 Clinical Ethics Unit, University Hospital of Basel and Psychiatric University Clinics Basel, Basel, Switzerland; 4 Department of Health Professions, School of Nursing, Bern University of Applied Sciences, Bern, Switzerland; 5 Department of Obstetrics and Gynecology, University Hospital of Bern, Bern, Switzerland

**Correction to: BMC Pregnancy Childbirth 21, 369 (2021)**

**https://doi.org/10.1186/s12884-021-03826-1**

Following publication of the original article [1], the authors identified an error in the author name of Eva Cignacco.

The incorrect author name is: Eva Cignacco Müller

The correct author name is: Eva Cignacco

The original article [1] has been updated.
