# Supplementary material for: Informal coercion during childbirth: risk factors and prevalence estimates from a nationwide survey of women in Switzerland
Source: BMC Pregnancy Childbirth. 2021 May 10;21:369. doi: 10.1186/s12884-021-03826-1 (PMC8112037; doi:10.1186/s12884-021-03826-1)
Supplement: Supplementary file 1 — Additional file 1: Table S1a-d. List of independent variables, item wording (in English, German, French, and Italian), sources and possible transformations. [file 12884_2021_3826_MOESM1_ESM.docx]

Additional File 1

[Table S1a. List of independent variables, item wording (English), sources and possible transformations. 2](#_Toc67977964)

[Table S1b. List of independent variables, item wording (German). 16](#_Toc67977965)

[Table S1c. List of independent variables, item wording (French). 26](#_Toc67977966)

[Table S1d. List of independent variables, item wording (Italian). 36](#_Toc67977967)

# Table S1a. List of independent variables, item wording (English), sources and possible transformations.

| **Variable** | **Source / question** | **Response options / scale** | **Transformations / comments** |
| --- | --- | --- | --- |
| Birth preparation | How did you **prepare for the birth**? Multiple answers are possible. | - Antenatal classes - With books - Online - By writing a birth plan - No special preparation - Other: ______ | Based on “other” responses, 9 new categories were built: *conversations* (with friends, HCP, etc.), *techniques* (hypnobirthing, meditation, visualization, etc.), *alternative therapies* (acupuncture, homeopathy, etc.), *information* (videos, apps, tv, social media), *yoga*, *sports*, *perineum preparation*, *diet* and *other*.  To reflect the amount of individual birth preparation, the sum of all categories selected by a respondent and a “yes” answer to the next question on considering various birth place options were entered in the model. |
|  | Did you consider various options before deciding on this place of birth? | - Yes - No |  |
| Main caregiver pregnancy | Which **specialist** was your main caregiver **during your pregnancy?** | - a doctor - a midwife - Other: ______ |  |
| High-risk pregnancy | Did you require medical treatment during your pregnancy? | - Yes, I was admitted to the hospital as an in-patient (overnight stay) - Yes, as an out-patient - No |  |
| Child’s age | How old is your child now? | 14 choices: “less than 1 month”, “1 month” up to “12 months”, “more than 12 months” |  |
| Child’s weight | How much did your child weigh at birth **approximately**? | - in grams (g) | (accepting numerical value between 500 and 6000 grams) |
| Gestational age | At how many **weeks of pregnancy** was your child born? | - < 32 (more than 8 weeks early) - 32-36 (more than 3 weeks early) - 37 - 41 - > 41 - I am not sure |  |
| Multiple birth | Was this a multiple birth? | - Yes, I had twins, triplets, etc. - No |  |
| Time of birth | At about what time was your child born? | Slider bar, labelled from left to right with:   \| at night \|  \| in the morning \|  \| at noon \|  \| in the evening \|  \| at night \| \| --- \| --- \| --- \| --- \| --- \| --- \| --- \| --- \| --- \| \| 0:00 \| 3:00 \| 6:00 \| 9:00 \| 12:00 \| 15:00 \| 18:00 \| 21:00 \| 24:00 \| | |
| NICU transfer | Did your child require treatment in the neonatal intensive care unit (NICU) or the neonatology unit after birth? | - Yes - No |  |
| Nulliparous | Was this your first birth? | - Yes - No |  |
| Previous CS | Did you have a caesarean section for a previous birth? | - Yes - No |  |
| Place of birth | Where did you give birth? | - University hospital - Cantonal hospital - Regional hospital - Private hospital - Birthing centre - At home - Other: ____________ | “University hospital”, “Cantonal hospital” and “Regional hospital” were combined to “Public hospital” for statistical analyses. |
| Planned place of birth | You specified that you gave birth at [previous answer]. Is this where you were planning to give birth? | - Yes - No, the original plan was to give birth at: ________ |  |
| Known care provider | Did any of the health professionals who looked after you during labour also care for you during your pregnancy? | - Yes - No |  |
| Accompaniment | Who accompanied you at this birth? Multiple answers are possible. | - Husband/life partner - Family member - Doula - Friend - Nobody - Other: ____________ | As 98% of women were accompanied by their husband/life partner, the variation in this variable was low and it was therefore excluded from statistical analyses. |
| Preference for shared decision making | From your point of view, who should have made important decisions about how to proceed during childbirth?   - I should have made the decision on my own after I had been fully informed. - The health professional should have made the decision on my behalf after I had been fully informed. - I should have made the decision together with the health professional after I had been fully informed. | Likert scale:   - Strongly agree - Agree - Neither agree nor disagree - Disagree - Strongly disagree | Optimal scaling procedure and subsequent PCA. |
| Atmosphere | To what extent would you agree with the following statements regarding the birth?   - I felt that the health professionals were open to my wishes and needs. - The health professionals and I made the important decisions together. - I felt that the midwives and doctors worked well together. | Likert scale:   - Strongly agree - Agree - Neither agree nor disagree - Disagree - Strongly disagree | Chi-square tests between these single items and the item “pressure” revealed maximal odds ratios of 17.3 [95% CI 13.1, 22.72], suggesting that the variable “atmosphere” serves as a proxy rather than a predictor for pressure. It was therefore excluded from final analyses. |
| Birth mode preference | What kind of birth did you want **while you were pregnant**? | Visual analog scale, ranging from “Definitely a spontaneous vaginal delivery” (left) to “Definitely a caesarean section” (right) |  |
| Birth mode | How did you actually give birth? | - Spontaneous vaginal delivery - Forceps or vacuum delivery - Caesarean section |  |
|  | You stated that you had a caesarean section. **When** was it decided that you should have a caesarean section? | - The caesarean section was already planned before going to the hospital. - After delivery had already begun. - I required an emergency caesarean after less than 15 minutes. | Coded as “planned CS”, “unplanned CS” and “emergency CS” |
| Indication Planned CS | What was the reason you had a caesarean? Multiple answers are possible. | - I had health problems (e.g. preeclampsia). - Caesarean during a previous birth - Estimated weight of child at birth - Position/presentation of the baby (e.g. breech position) - Multiple birth (twins, triplets etc.) - The child was unwell. - It’s what I wanted. - I didn’t understand the reason. - I can’t remember. - Other reasons: _________________ |  |
| Reason elective CS | Why was it you wanted a caesarean section? Multiple answers are possible. | - Fear of pain or complications - Fear for my child’s safety - Earlier negative birth experience - Ability to plan the birth - To avoid injuries in the genital area - Other reasons: ________________ |  |
| Indication unplanned CS | What was the reason you had a caesarean? Multiple answers are possible. | - Health problems. - Prolonged labour/failure to progress - Position/presentation of the child - Failed induction of labour. - The child was unwell. - The pain was too severe. - I was exhausted. - I didn’t understand the reason. - I can’t remember. - Other reasons: _________________ |  |
| Indication instrumental vaginal birth | Why was it you had a forceps or vacuum delivery? Multiple answers are possible. | - The child was unwell. - Prolonged labour/failure to progress - I was exhausted. - I didn’t understand the reason. - I can’t remember. - Other reasons: _________________ |  |
| Duration of birth | **Roughly** how many hours were you in labour **after your contractions became regular**? | Slider bar, ranging from “0” (left) to “48+” (right). |  |
| Interventions | What (other) procedures were performed during the birth? Multiple answers are possible. | - Induction of labour - Vaginal examination - Electronic monitoring of contractions and heartbeat (CTG) - Episiotomy (surgical cut to enlarge opening of vagina) - Manual pressure on the abdomen to speed up birth - Rupture of membranes to release amniotic fluid (“breaking the water”) - I can’t remember. - None - Other procedures: _________________ |  |
| CTG | You indicated that monitoring of your **child’s heartbeat and your contractions (CTG)** was applied. Did you find the CTG uncomfortable? | - Yes - No |  |
| Vaginal examinations | Which of the following statements apply to the **vaginal examination(s)**?   - They did everything to make the examination bearable. - I was examined too often. - My privacy was respected. | Likert scale:   - Strongly agree - Agree - Neither agree nor disagree - Disagree - Strongly disagree |  |
| Indication induction of labor | Why was it that **labour was induced** in your case? Multiple answers are possible. | - The baby was unwell. - Gestational diabetes - I had other health problems (e.g. high blood pressure, preeclampsia). - Overdue/prolonged pregnancy - The child was considered too large. - Premature rupture of membranes and risk of infection - There were problems with the amniotic fluid (“the waters”). - My baby did not grow as expected. - I didn’t understand the reason. - I can’t remember. - Other reasons: _________________ |  |
| Indication episiotomy | Why was it you had an **episiotomy**? Multiple answers are possible. | - The child was unwell. - The baby was too big. - To avoid more severe injuries in the genital area. - Prolonged labour/failure to progress - I didn’t understand the reason. - I can’t remember. - Other reasons: _________________ |  |
| Indication amniotomy | Why was it you had **induced rupture of your membranes**? Multiple answers are possible. | - To speed up the birth - The child was unwell. - I didn’t understand the reason. - I can’t remember. - Other reasons: _________________ |  |
| Medication | Which medications or painkillers did you receive? Multiple answers are possible. | - Epidural anaesthesia (EDA, pain relief administered via the spine) - Patient-controlled button for analgesia (PCA pump) - Laughing gas - Other painkillers - Labour-inducing drugs - Labour-inhibiting drugs - Antibiotics - I receive some medication, but I’m not sure what it was. - None - Other: _________________ |  |
| Freedom of movement (CS) | Before you had to have a caesarean section, were you able to move around and choose the position you found most comfortable during the birth? | - Yes - No, because of the epidural anaesthesia (EDA) - No, because of the CTG (foetal monitoring). - No, for other reasons: _________________ |  |
| Freedom of movement (vaginal birth) | Were you able to move around and choose the position you found most comfortable during the birth? Multiple answers are possible. | - Yes - No, because of the epidural anaesthesia (EDA) - No, because of the CTG (foetal monitoring). - No, for other reasons: _________________ |  |
| Denied procedures | Were you **denied** any procedures (examinations, treatments) during the birth, even though you expressly requested them? | - No - Yes, namely: _________________ |  |
| Postnatal debriefing | **After you gave birth**, did you have the opportunity to discuss the birth with the health professionals involved? | - Yes - I didn’t feel the need. - No, because: _________________ |  |
| Postnatal debriefing helpful | Did this follow-up interview help you clear up or process something important? | - Yes - No |  |
| Maternal age | How old are you? | - 18-23 - 24-27 - 28-31 - 32-35 - 36-39 - 40+ |  |
| Nationality | What is your nationality? | - Swiss - German - French - Italian - Austrian - Afghan - Bosnian/Herzegovian - Brazilian - Chinese - Croatian - Eritrean - Hungarian - Kosovan - Macedonian - Polish - Portuguese - Romanian - Russian - Serbian - Slovakian - Spanish - Sri Lankan - Syrian - Turkish - US citizen - UK citizen - Other | “German”, “French”, “Italian” and “Austrian” were combined to “Neighboring state”, all other non-Swiss nationalities were combined to “other”. |
| Birth canton | In which canton was your child born? | - Aargau - Appenzell Ausserrhoden - Appenzell Innerrhoden - Basel-Landschaft - Basel-Stadt - Berne - Freiburg - Geneva - Glarus - Grisons - Jura - Lucerne - Neuenburg - Nidwalden - Obwalden - Schaffhausen - Schwyz - Solothurn - St. Gallen - Ticino - Thurgau - Uri - Vaud - Wallis - Zug - Zürich |  |
| Urbanization | Federal Statistical Office (FSO), [www.bfs.admin.ch](http://www.bfs.admin.ch) , Structure of the permanent resident population by canton, 1999-2018 |  | Based on the canton where the child was born (previous question), we used the percentage of permanent residents living in an “urban core area” or “area influenced by urban cores” as an indicator of urbanization within each canton. |
| Marital status | What is your marital status? | - Married/civil partnership - Single - Divorced - Widowed | Because <2% were either divorced or widowed, we combined them with the category “single”. |
| Socio-economic status | What is your net monthly **household** income? (the current income of all household members **together**) | - less than CHF 3,000 - CHF 3,000 – 4,999 - CHF 5,000 – 6,999 - CHF 7,000 – 8,999 - CHF 9,000 – 11,999 - CHF 12,000 – 15,000 - more than CHF 15,000 | Questions on income and mother’s education were reduced to socio-economic status using optimal scaling procedure and subsequent PCA. |
|  | What is your level of education or training? | - Compulsory education - Apprenticeship - Grammar school, vocational A level, specialized secondary school certificate (FMS), vocational college (DMS) - Higher technical and vocational training - University of Applied Sciences, educational college - University, Federal Institute of Technology (EPFL, ETH) - Other: __________________ |  |
| Health insurance | How are you insured? | - General - Semi-private - Private - I don’t know | Categories semi-private and private were combined to one category. |

# Table S1b. List of independent variables, item wording (German).

| **Variable** | **Source / question** | **Response options / scale** |
| --- | --- | --- |
| Birth preparation | Wie haben Sie sich **auf die Geburt vorbereitet**? Es sind mehrere Antworten möglich. | - Geburtsvorbereitungskurs - mit Büchern - im Internet - Erstellen eines Geburtsplans - keine spezielle Vorbereitung - Anderes: ______ |
|  | Haben Sie sich mit verschiedenen Möglichkeiten auseinandergesetzt, bevor Sie sich für diesen Geburtsort entschieden haben? | - Ja - Nein |
| Main caregiver pregnancy | Welche **Fachperson** hat Sie **während der Schwangerschaft** hauptsächlich betreut? Es sind mehrere Antworten möglich. | - Arzt/Ärztin - Hebamme - Andere: ______ |
| High-risk pregnancy | Mussten Sie **in der Schwangerschaft medizinisch behandelt** werden? Es sind mehrere Antworten möglich. | - Ja, stationär im Spital (mit Übernachtung) - Ja, ambulant - Nein |
| Child’s age | Wie **alt** ist Ihr Kind heute? | 14 choices: “weniger als 1 Monat”,“1 Monat” up to “12 Monate”, “älter als 12 Monate” |
| Child’s weight | Wie **schwer** war Ihr Kind bei der Geburt ungefähr? | - in Gramm (g) |
| Gestational age | In welcher **Schwangerschaftswoche** wurde Ihr Kind geboren? | - < 32 (mehr als 8 Wochen zu früh) - 32 - 36 (mehr als 3 Wochen zu früh) - 37 - 41 - > 41 - Ich bin mir nicht sicher |
| Multiple birth | Handelt es sich bei dieser Geburt um eine **Mehrlingsgeburt**? | - Ja, Zwillinge, Drillinge etc. - Nein |
| Time of birth | Um welche **Zeit** kam Ihr Kind ungefähr zur Welt? | Slider bar, labelled from left to right with:   \| Nacht \|  \| Morgen \|  \| Mittag \|  \| Abend \|  \| Nacht \| \| --- \| --- \| --- \| --- \| --- \| --- \| --- \| --- \| --- \| \| 0:00 \| 3:00 \| 6:00 \| 9:00 \| 12:00 \| 15:00 \| 18:00 \| 21:00 \| 24:00 \| |
| NICU transfer | Wurde Ihr Kind nach der Geburt auf der Kinderintensivstation respektive Neonatologie behandelt? | - Ja - Nein |
| Nulliparous | War dies Ihre **erste Geburt**? | - Ja - Nein |
| Previous CS | Wurde bei einer **früheren Geburt** ein Kaiserschnitt durchgeführt? | - Ja - Nein |
| Place of birth | Wo haben Sie geboren? | - Universitätsspital - Kantonsspital - Regionalspital - Privatspital - Geburtshaus - Zuhause - Anderes: ____________ |
| Planned place of birth | Sie haben als Geburtsort [vorherige Antwort] angegeben. War dies von Anfang an so geplant? | - Ja - Nein, geplant war die Geburt hier: ________ |
| Known care provider | Hat Sie eine der Fachpersonen, die Sie **unter der Geburt begleitete**, bereits **während der Schwangerschaft** betreut? | - Ja - Nein |
| Accompaniment | Wer hat Sie bei dieser Geburt begleitet? Es sind mehrere Antworten möglich. | - Ehemann / Lebenspartner-in - Familienmitglied - Doula - Freundin/Freund - Keine Begleitung - Other: ____________ |
| Preference for shared decision making | Wer hätte **aus Ihrer Sicht** wichtige Entscheidungen zum weiteren Vorgehen unter der Geburt fällen sollen?   - Ich hätte die Entscheidung **allein** treffen sollen, nachdem ich umfassend informiert wurde. - Die **Fachperson** hätte die Entscheidung **für mich** treffen sollen, nachdem ich vollständig informiert wurde. - Ich hätte die Entscheidung **zusammen** mit der Fachperson treffen sollen, nachdem ich vollständig informiert wurde. | Likert scale:   - Stimme voll zu - Stimme zu - Stimme weder zu noch nicht zu - Stimme nicht zu - Stimme überhaupt nicht zu |
| Atmosphere | Inwiefern können Sie folgenden Aussagen in Bezug auf Ihre Geburt zustimmen?   - Ich hatte den Eindruck, dass die Fachpersonen gegenüber meinen Wünschen und Bedürfnissen offen waren. - Das Fachpersonal und ich haben wichtige Entscheidungen gemeinsam gefällt. - Ich hatte den Eindruck, dass die Zusammenarbeit zwischen den Hebammen und den Ärztinnen oder Ärzten gut funktioniert. | Likert scale:   - Stimme voll zu - Stimme zu - Stimme weder zu noch nicht zu - Stimme nicht zu - Stimme überhaupt nicht zu |
| Birth mode preference | Was für eine Geburt haben Sie sich **in der Schwangerschaft** gewünscht? | Visual analog scale, ranging from “sicher eine vaginale/spontane Geburt” (left) to “sicher einen Kaiserschnitt” (right) |
| Birth mode | Wie haben Sie nun entbunden? | - vaginal/spontane Geburt - Saugglocken-/Vakuum- oder Zangengeburt - Kaiserschnitt |
|  | Sie haben angegeben, dass bei Ihnen ein Kaiserschnitt durchgeführt wurde. **Wann** fiel die Entscheidung, einen Kaiserschnitt durchzuführen? | - Der Kaiserschnitt war bereits vor Eintritt ins Spital so geplant. - Nach bereits begonnener Geburt. - Der Kaiserschnitt musste notfallmässig innerhalb von 15 Minuten durchgeführt werden. |
| Indication Planned CS | Wieso wurde ein Kaiserschnitt durchgeführt? Es sind mehrere Antworten möglich. | - Ich hatte gesundheitliche Probleme (z.B. Schwangerschaftsvergiftung). - Kaiserschnitt bei früherer Geburt. - geschätztes kindliches Geburtsgewicht. - Lage/Position des Kindes (z.B. Beckenendlage). - Mehrlinge (Zwilling, Drillinge etc.). - Dem Kind ging es nicht gut. - Es war mein eigener Wunsch. - Ich habe den Grund nicht verstanden. - Ich kann mich nicht erinnern. - Andere Gründe: ________ |
| Reason elective CS | Wieso wünschten Sie einen Kaiserschnitt? Es sind mehrere Antworten möglich. | - Angst vor Schmerzen oder Komplikationen - Angst um Sicherheit des Kindes - Früheres negatives Geburtserlebnis - Planbarkeit der Geburt - Um Verletzungen im Genitalbereich zu vermeiden - Andere Gründe: _________ |
| Indication unplanned CS | Wieso wurde ein Kaiserschnitt durchgeführt?  Es sind mehrere Antworten möglich | - Ich hatte gesundheitliche Probleme. - Fehlender Geburtsfortschritt - Lage/Position des Kindes - Die Geburtseinleitung war nicht wirksam. - Dem Kind ging es nicht gut. - Die Schmerzen waren zu gross. - Ich war erschöpft. - Ich habe den Grund nicht verstanden. - Ich kann mich nicht erinnern. - Andere Gründe: _________ |
| Indication instrumental vaginal birth | Wieso wurde eine Saugglocken-/Vakuum- oder Zangengeburt durchgeführt? Es sind mehrere Antworten möglich. | - Dem Kind ging es nicht gut. - Fehlender Geburtsfortschritt. - Ich war erschöpft. - Ich habe den Grund nicht verstanden. - Ich kann mich nicht erinnern. - Andere Gründe: _________ |
| Duration of birth | Wie viele Stunden hat die Geburt **ab Beginn regelmässiger Wehen ungefähr gedauert**? | Slider bar, ranging from “0” (left) to “48+” (right). |
| Interventions | Welche (weiteren) Massnahmen wurden unter der Geburt durchgeführt? Es sind mehrere Antworten möglich. | - Geburtseinleitung - Vaginale Untersuchungen - Elektronische Überwachung der Wehen und Herztöne (CTG) - Dammschnitt zur Erweiterung des Scheideneingangs (Episiotomie) - Druck auf den Bauch zur Beschleunigung der Geburt - Eröffnen der Fruchtblase - Ich kann mich nicht erinnern - Keine - Andere Massnahmen: ________ |
| CTG | Sie haben angewählt, dass bei Ihnen die kindlichen Herztöne und Ihre Wehen mittels CTG gemessen wurden. Fühlten Sie sich durch das CTG gestört? | - Ja - Nein |
| Vaginal examinations | Welche Aussagen treffen auf die **vaginalen Untersuchungen** zu?   - Es wurde alles getan, um die Untersuchungen erträglich zu machen. - Ich wurde zu häufig untersucht. - Meine Intimsphäre wurde gewahrt. | Likert scale:   - Stimme voll zu - Stimme zu - Stimme weder zu noch nicht zu - Stimme nicht zu - Stimme überhaupt nicht zu |
| Indication induction of labor | Wieso wurde bei Ihnen **die Geburt eingeleitet**? Es sind mehrere Antworten möglich. | - Dem Kind ging es nicht gut. - Diabetes in der Schwangerschaft (Zuckerkrankheit) - Ich hatte andere gesundheitliche Probleme (z.B. Bluthochdruck, Schwangerschaftsvergiftung). - Terminüberschreitung, Übertragung - Das Kind wurde als zu gross eingeschätzt. - Vorzeitiger Blasensprung und Angst vor Infektionen - Es gab Probleme mit dem Fruchtwasser - Mein Kind ist nicht genügend gewachsen. - Ich habe den Grund nicht verstanden - Ich kann mich nicht erinnern. - Andere Gründe: _________ |
| Indication episiotomy | Wieso wurde bei Ihnen **ein Dammschnitt** durchgeführt? Es sind mehrere Antworten möglich. | - Dem Kind ging es nicht gut. - Das Kind war zu gross. - Vermeiden von grösseren Verletzungen im Genitalbereich. - Fehlender Geburtsfortschritt. - Ich habe den Grund nicht verstanden. - Ich kann mich nicht erinnern. - Andere Gründe: __________ |
| Indication amniotomy | Wieso wurde bei Ihnen **die Fruchtblase eröffnet**? Es sind mehrere Antworten möglich. | - Beschleunigung der Geburt - Dem Kind ging es nicht gut - Ich habe den Grund nicht verstanden - Ich kann mich nicht erinnern. - Andere Gründe: __________ |
| Medication | Welche Medikamente oder Schmerzmittel haben Sie erhalten? Es sind mehrere Antworten möglich. | - PDA, EDA (Schmerzlinderung über Rückenmark) - Schmerzmittelpumpe (PCA, Happybutton) - Lachgas - Andere Schmerzmittel - Wehenförderndes Mittel - Mittel zur Hemmung der Wehen - Antibiotika - Ich habe ein Medikament erhalten, weiss aber nicht was es war. - Keine - Andere: __________ |
| Freedom of movement (CS) | Konnten Sie sich, bevor der Kaiserschnitt gemacht werden musste, während der Geburt bewegen und die Positionen frei wählen? Es sind mehrere Antworten möglich. | - Ja - Nein, wegen der PDA (Schmerzlinderung über Rückenmark). - Nein, wegen dem CTG (elektronische Überwachung). - Nein, aus anderen Gründen: __________ |
| Freedom of movement (vaginal birth) | Konnten Sie sich während der Geburt bewegen und die Positionen frei wählen? Es sind mehrere Antworten möglich. | - Ja - Nein, wegen der PDA (Schmerzlinderung über Rückenmark). - Nein, wegen dem CTG (elektronische Überwachung). - Nein, aus anderen Gründen: __________ |
| Denied procedures | Wurden Ihnen unter der Geburt **Massnahmen (Untersuchungen, Behandlungen) verweigert**, obwohl Sie sich diese ausdrücklich gewünscht haben? | - Nein - Ja, folgende Massnahmen: ______ |
| Postnatal debriefing | Hatten Sie **nach der Geburt** die Möglichkeit, mit den **beteiligten Fachpersonen** Ihre Geburt zu **besprechen**? | - Ja - Ich hatte kein Bedürfnis. - Nein, weil: _________________ |
| Postnatal debriefing helpful | Hat Ihnen dieses Nachgespräch geholfen, wichtige Dinge zu klären oder zu verarbeiten? | - Ja - Nein |
| Maternal age | Wie **alt** sind Sie? | - 18-23 - 24-27 - 28-31 - 32-35 - 36-39 - 40+ |
| Nationality | Welcher **Nationalität** gehören Sie an? | - Schweiz - Deutschland - Frankreich - Italien - Österreich - Afghanistan - Afrika - Asien - Belgien - Bosnien und Herzegowina - Brasilien - China - Eritrea - Kosovo - Kroatien - Mazedonien - Österreich - Polen - Portugal - Rumänien - Russland - Serbien - Slowakei - Spanien - Sri Lanka - Südamerika - Syrien - Türkei - Ungarn - Vereinigte Staaten - Vereinigtes Königreich - Andere |
| Birth canton | In welchem Kanton kam **Ihr Kind** zur Welt? | - Aargau - Appenzell Ausserrhoden - Appenzell Innerrhoden - Basel-Landschaft - Basel-Stadt - Bern - Freiburg - Genf - Glarus - Graubünden - Jura - Luzern - Neuenburg - Nidwalden - Obwalden - Schaffhausen - Schwyz - Solothurn - St. Gallen - Tessin - Thurgau - Uri - Waadt - Wallis - Zug - Zürich |
| Marital status | Welchen **Zivilstand** haben Sie? | - verheiratet / eingetragene Partnerschaft - ledig - geschieden - verwitwet |
| Socio-economic status | Wie hoch ist Ihr **monatliches Haushalts**einkommen ungefähr? (das gegenwärtige Einkommen aller Haushaltsmitglieder **zusammen**) | - weniger als 3'000 CHF - 3'000 – 4’999 CHF - 5'000 – 6’999 CHF - 7'000 – 8’999 CHF - 9'000 – 11’999 CHF - 12'000 – 15’000 CHF - mehr als 15'000 CHF |
|  | Welche Schule oder Ausbildung haben Sie abgeschlossen? | - obligatorische Schule - Berufslehre - Gymnasium, Berufsmatura, FMS, DMS - Höhere Fachs- und Berufsbildung - Fachhochschule, PH - Universität, ETH, EPFL - Andere: __________________ |
| Health insurance | Wie sind sie versichert? | - allgemein - halbprivat - privat - Ich weiss es nicht |

# Table S1c. List of independent variables, item wording (French).

| **Variable** | **Source / question** | **Response options / scale** |
| --- | --- | --- |
| Birth preparation | Comment vous êtes-vous **préparée à l’accouchement**? Plusieurs réponses sont possibles. | - Cours de préparation à l’accouchement - Avec des livres - Sur Internet - Établissement d’un plan de naissance - Pas de préparation particulière - Autre:______ |
|  | Avez-vous étudié différentes possibilités avant de choisir ce lieu d’accouchement? | - Oui - Non |
| Main caregiver pregnancy | Quel spécialiste a réalisé la plus grande partie de votre suivi **pendant la grossesse**? Plusieurs réponses sont possibles. | - Médecin - Sage-femme - Autre:______ |
| High-risk pregnancy | Avez-vous dû suivre un **traitement médical pendant la grossesse**? Plusieurs réponses sont possibles. | - Oui, pendant une hospitalisation (avec nuitée) - Oui, en ambulatoire - Non |
| Child’s age | Quel est l’âge de votre enfant aujourd’hui? | 14 choices: “< 1 mois”,“1 mois” up to “12 mois”, “plus de 12 Mois” |
| Child’s weight | Quel était le poids environ de votre enfant à la naissance? | - en grammes (g) |
| Gestational age | Lors de quelle **semaine de grossesse** votre enfant est-il né? | - Avant la semaine 32 (plus de 8 semaines trop tôt) - 32 - 36 (plus de 3 semaines trop tôt) - 37 - 41 - Après la semaine 41 - Je ne suis pas sûre |
| Multiple birth | S’agit-il d’une **naissance multiple**? | - Oui, jumeaux, triplés, etc. - Non |
| Time of birth | À quelle heure votre enfant est-il né? | Slider bar, labelled from left to right with:   \| la nuit \|  \| le matin \|  \| à midi \|  \| le soir \|  \| la nuit \| \| --- \| --- \| --- \| --- \| --- \| --- \| --- \| --- \| --- \| \| 0:00 \| 3:00 \| 6:00 \| 9:00 \| 12:00 \| 15:00 \| 18:00 \| 21:00 \| 24:00 \| |
| NICU transfer | Après la naissance, votre enfant a-t-il été traité dans une unité de soins intensifs pédiatriques ou en néonatologie? | - Oui - Non |
| Nulliparous | Était-ce votre premier accouchement? | - Oui - Non |
| Previous CS | Une césarienne a-t-elle été pratiquée lors d’**un accouchement précédent**? | - Oui - Non |
| Place of birth | Où avez-vous accouché? | - Hôpital universitaire - Hôpital cantonal - Hôpital régional - Hôpital privé - Maison de naissance - À domicile - Autre: __________________ |
| Planned place of birth | Vous avez indiqué [réponse précédente] comme lieu d’accouchement. Était-ce prévu dès le départ? | - Oui - Non, le lieu prévu était: _______ |
| Known care provider | Parmi les spécialistes qui vous ont **accompagnée** **lors de l’accouchement**, l’un / l’une d’entre eux vous avait-il **également** suivie **pendant la grossesse**? | - Oui - Non |
| Accompaniment | Qui vous a accompagnée lors de cet accouchement?  Plusieurs réponses sont possibles. | - Époux / partenaire - Membre de la famille - Doula - Amie/ami - Pas d’accompagnement - Autre: ____________ |
| Preference for shared decision making | **D’après vous**, qui aurait dû prendre les décisions importantes relatives à la procédure lors de l’accouchement?   - J'aurais dû décider **seule** après avoir obtenu des informations complètes. - Le **spécialiste** aurait dû décider **pour moi** une fois m’avoir donné des informations complètes. - J'aurais dû décider **avec le spécialiste** après avoir obtenu des informations complètes. | Likert scale:   - Tout à fait d’accord - D’accord - Ni d’accord, ni pas d’accord - Pas d’accord - Pas du tout d’accord |
| Atmosphere | Dans quelle mesure êtes-vous d’accord avec les affirmations suivantes **en lien avec votre accouchement**?   - J’ai eu l’impression que les spécialistes étaient ouverts à mes souhaits et besoins. - Les spécialistes et moi avons pris les décisions importantes ensemble. - J’ai eu l’impression que la collaboration entre les sages-femmes et les médecins fonctionnait bien. | Likert scale:   - Tout à fait d’accord - D’accord - Ni d’accord, ni pas d’accord - Pas d’accord - Pas du tout d’accord |
| Birth mode preference | Quelle naissance souhaitiez-vous **pendant la grossesse**? | Visual analog scale, ranging from “Clairement un accouchement spontané” (left) to “Clairement une césarienne” (right) |
| Birth mode | Comment avez-vous réellement accouché? | - Accouchement spontané par voie basse - Accouchement avec ventouse/aspiration ou forceps - Césarienne |
|  | Vous avez indiqué avoir subi **une césarienne**. **Quand** la décision de pratiquer une césarienne a-t-elle été prise? | - La césarienne était déjà prévue avant l’entrée à l’hôpital. - Après le début de l’accouchement. - La césarienne a dû être pratiquée d’urgence dans un délai de 15 minutes. |
| Indication Planned CS | **Pourquoi une césarienne** a-t-elle été pratiquée? Plusieurs réponses sont possibles. | - J’ai eu des problèmes de santé (par ex. pré-éclampsie) - Césarienne lors d’une grossesse précédente - Estimation du poids de l’enfant à la naissance - Emplacement/position de l’enfant (par ex. présentation par le siège) - Naissance multiple (jumeaux, triplés etc.) - L’enfant n’allait pas bien. - C’était mon propre souhait. - Je n’ai pas compris la raison. - Je ne me souviens plus. - Autres raisons: ________ |
| Reason elective CS | Pourquoi **vouliez-vous** une césarienne? Plusieurs réponses sont possibles. | - Peur de la douleur ou des complications. - Peur pour la sécurité de l’enfant. - Expérience d’accouchement précédente négative. - Possibilité de planifier la naissance. - Prévention de lésions dans la région génitale - Autres raisons: ___________ |
| Indication unplanned CS | Pourquoi une **césarienne** a-t-elle été pratiquée? Plusieurs réponses sont possibles. | - J’avais des problèmes de santé. - L’accouchement ne progressait pas. - Emplacement/position de l’enfant. - Le déclenchement du travail a été inefficace. - L’enfant n’allait pas bien. - La douleur était trop forte. - J’étais épuisée. - Je n’ai pas compris la raison. - Je ne me souviens plus. - Autres raisons: _________ |
| Indication instrumental vaginal birth | Pourquoi un accouchement avec **ventouse/aspiration ou forceps** a-t-il été pratiqué? Plusieurs réponses sont possibles. | - L’enfant n’allait pas bien. - L’accouchement ne progressait pas. - J’étais épuisée. - Je n’ai pas compris la raison. - Je ne me souviens plus. - Autres raisons: |
| Duration of birth | Combien d’heures **environ** a duré l’accouchement **à compter des contractions régulières**? | Slider bar, ranging from “0” (left) to “48+” (right). |
| Interventions | Quelles (autres) mesures ont été pratiquées lors de l’accouchement? Plusieurs réponses sont possibles. | - Déclenchement du travail - Examens gynécologiques - Surveillance électronique des contractions et du rythme cardiaque (CTG) - Épisiotomie (coupure chirurgicale pour élargir l'ouverture du vagin) - Pression sur le ventre pour accélérer l’accouchement - Rupture de la poche des eaux - Je ne me souviens plus - Aucune - Autres mesures:________ |
| CTG | Vous avez indiqué que **les bruits et contractions** cardiaques de votre enfant ont été mesurés par la **CTG**. Avez-vous été gênée par la CTG? | - Oui - Non |
| Vaginal examinations | Quelles affirmations correspondent à votre situation concernant les **examens vaginaux**?   - Tout a été fait pour rendre les examens supportables. - J’ai été examinée trop souvent. - Mon intimité a été préservée. | Likert scale:   - Tout à fait d’accord - D’accord - Ni d’accord, ni pas d’accord - Pas d’accord - Pas du tout d’accord |
| Indication induction of labor | Pourquoi l’**accouchement a-t-il été déclenché** chez vous? Plusieurs réponses sont possibles. | - L’enfant n’allait pas bien. - Diabète gestationnel - J’ai eu d’autres problèmes de santé (par ex. hypertension, pré-éclampsie). - Grossesse prolongée, dépassement du terme - L’enfant a été jugé trop grand. - Rupture prématurée de la poche des eaux et peur des infections. - Il y a eu des problèmes avec le liquide amniotique. - Mon enfant n’a pas assez grandi. - Je n’ai pas compris la raison. - Je ne me souviens plus. - Autres raisons:_________ |
| Indication episiotomy | Pourquoi une **épisiotomie** a-t-elle été pratiquée? Plusieurs réponses sont possibles. | - L’enfant n’allait pas bien. - L’enfant était trop grand. - Prévention de lésions plus graves dans la région génitale. - L’accouchement ne progressait pas. - Je n’ai pas compris la raison. - Je ne me souviens plus. - Autres raisons:__________ |
| Indication amniotomy | Pourquoi votre **poche des eaux a-t-elle été rompue**? Plusieurs réponses sont possibles. | - Pour accélérer l’accouchement. - L’enfant n’allait pas bien. - Je n’ai pas compris la raison. - Je ne me souviens plus. - Autres raisons: _________ |
| Medication | Quels médicaments ou antidouleurs avez-vous reçus? Plusieurs réponses sont possibles. | - Péridurale, épidurale (apaisement de la douleur par la moelle épinière) - Pompe à antalgiques (ACP, Happy Button) - Gaz hilarant - Autres antidouleurs - Produit stimulant les contractions - Produit pour inhiber les contractions - Antibiotiques - On m’a donné un médicament, mais je ne sais pas de quoi il s’agissait - Aucune - Autre: __________ |
| Freedom of movement (CS) | Avant que la césarienne ne doive être pratiquée, avez-vous pu bouger et choisir vos positions pendant l’accouchement ? Plusieurs réponses sont possibles. | - Oui - Non, à cause de la péridurale. - Non, à cause de la CTG (surveillance électronique). - Non, pour d’autres raisons: __________ |
| Freedom of movement (vaginal birth) | Avez-vous pu bouger pendant l’accouchement et choisir librement les positions? Plusieurs réponses sont possibles. | - Oui - Non, à cause de la péridurale. - Non, à cause de la CTG (surveillance électronique). - Non, pour d’autres raisons: __________ |
| Denied procedures | Des mesures vous ont-elles été **refusées** pendant l’accouchement (examens, traitements) alors que vous les aviez expressément demandées? | - Non - Oui, les mesures suivantes:______ |
| Postnatal debriefing | Après l’accouchement, avez-vous pu discuter de votre accouchement avec les spécialistes participants? | - Oui - Je n’en ai pas eu besoin. - Non, parce que: _________________ |
| Postnatal debriefing helpful | Cette discussion vous a-t-elle aidé à clarifier ou traiter des choses importantes? | - Oui - Non |
| Maternal age | Quel est **votre âge**? | - 18-23 - 24-27 - 28-31 - 32-35 - 36-39 - 40+ |
| Nationality | Quelle est **votre nationalité**? | - Suisse - Allemagne - France - Italie - Autriche - Afghanistan - Afrique - Asie - Belgique - Bosnie-Herzégovine - Brésil - Chine - Érythrée - Kosovo - Croatie - Macédoine - Autriche - Pologne - Portugal - Roumanie - Russie - Serbie - Slovaquie - Espagne - Sri Lanka - Amérique du Sud - Syrie - Turquie - Hongrie - États-Unis - Royaume-Uni - Autre |
| Birth canton | Dans quel canton **votre enfant** est-il né? | - Argovie - Appenzell Rhodes-Intérieures - Appenzell Rhodes-Extérieures - Bâle-Campagne - Bâle-Ville - Berne - Fribourg - Genève - Glaris - Grisons - Jura - Lucerne - Neuchâtel - Nidwald - Obwald - Schaffhouse - Schwyz - Soleure - Saint-Gall - Tessin - Thurgovie - Uri - Vaud - Valais - Zoug - Zurich |
| Marital status | Quel est votre **état civil**? | - mariée / partenariat enregistré - célibataire - divorcée - veuve |
| Socio-economic status | Quel est environ le revenu **mensuel** net de votre **foyer**? | - moins de 3'000 CHF - 3'000 – 4’999 CHF - 5'000 – 6’999 CHF - 7'000 – 8’999 CHF - 9'000 – 11’999 CHF - 12'000 – 15'000 CHF - plus de 15'000 CHF |
|  | Quel est votre niveau d'éducation ou de formation? | - École obligatoire - Apprentissage - Gymnase, maturité professionnelle, ECG, EDD - Formation spécialisée et professionnelle supérieure - Haute école spécialisée, HEP - Université, EPFL/ETH - Autre: __________________ |
| Health insurance | Quel est votre statut d’assurance? | - Assurance de base - Semi-privée - Privée - Je ne sais pas |

# Table S1d. List of independent variables, item wording (Italian).

| **Variable** | **Source / question** | **Response options / scale** |
| --- | --- | --- |
| Birth preparation | Come si è **preparata al parto**? Può fornire più risposte. | - Corso di preparazione al parto - Con libri - su internet - Creazione di un piano del parto - Nessuna preparazione particolare - Altro: ______ |
|  | Ha valutato diverse possibilità prima di scegliere questo luogo di nascita? | - Sì - No |
| Main caregiver pregnancy | Quale specialista L’ha seguita principalmente **durante la gravidanza**? Può fornire più risposte. | - Medico - Ostetrica/Levatrice - Altri: ______ |
| High-risk pregnancy | Ha dovuto sottoporsi a cure mediche in gravidanza? Può fornire più risposte. | - Sì, con ricovero ospedaliero (con pernottamento) - Sì, ambulatorialmente - No |
| Child’s age | Che età ha adesso il Suo bebè? | 14 choices: “meno di 1 mese”, “1 mese” up to “12 mesi”, “più di 12 mesi” |
| Child’s weight | Quanto pesava **approssimativamente** il bebè alla nascita? | - in grammi (g) |
| Gestational age | In quale **settimana di gravidanza** è nato? | - < 32 (più di 8 settimane prima del termine) - 32 - 36 (più di 3 settimane prima del termine) - 37 - 41 - > 41 - Non sono sicura |
| Multiple birth | Si è trattato di un parto gemellare? | - Sì, parto bigemino, trigemino ecc. - No |
| Time of birth | A che ora è nato il bebè? | Slider bar, labelled from left to right with:   \| Di notte \|  \| La mattina \|  \| A mezzogiorno \|  \| La sera \|  \| Di Notte \| \| --- \| --- \| --- \| --- \| --- \| --- \| --- \| --- \| --- \| \| 0:00 \| 3:00 \| 6:00 \| 9:00 \| 12:00 \| 15:00 \| 18:00 \| 21:00 \| 24:00 \| |
| NICU transfer | Il bebè, dopo il parto, ha avuto bisogno di cure nel reparto di terapia intensiva pediatrica o di neonatologia? | - Sì - No |
| Nulliparous | Era il Suo **primo parto**? | - Sì - No |
| Previous CS | In un **parto precedente** era stato effettuato un taglio cesareo? | - Sì - No |
| Place of birth | Dove ha partorito? | - Clinica universitaria - Ospedale cantonale - Ospedale regionale - Clinica privata - Casa maternità e nascita - A casa - Altro: ____________ |
| Planned place of birth | Ha dichiarato [risposta precedente] come luogo di nascita. Era stabilito fin dall'inizio? | - Sì - No, il luogo stabilito era: ________ |
| Known care provider | Uno degli specialisti che L’hanno seguita durante il parto L’aveva in cura anche durante la gravidanza? | - Sì - No |
| Accompaniment | Chi L’ha accompagnata in questo parto? Può fornire più risposte. | - Marito / compagno/a - Familiare - Amica/o - Doula - Senza accompagnamento - Altro: ____________ |
| Preference for shared decision making | Dal **Suo punto di vista**, chi avrebbe dovuto prendere decisioni importanti su come procedere durante il parto?   - Avrei dovuto decidere **da sola** dopo essere stata informata in modo esaustivo. - Lo **specialista** avrebbe dovuto decidere **per me** dopo avermi informata in modo esaustivo. - Avrei dovuto decidere **insieme allo specialista** dopo essere stata informata in modo esaustivo. | Likert scale:   - completamente d’accordo - d'accordo - né d’accordo né in disaccordo - in disaccordo - in completo disaccordo |
| Atmosphere | In che misura è d’accordo con le seguenti affermazioni **per quanto riguarda il Suo parto**?   - Ho avuto l’impressione che gli specialisti fossero aperti alle mie richieste e ai miei bisogni. - Gli specialisti e io abbiamo preso importanti decisioni insieme. - Ho avuto l’impressione che la collaborazione fra ostetriche/levatrice e medici funzionasse bene. | Likert scale:   - completamente d’accordo - d'accordo - né d’accordo né in disaccordo - in disaccordo - in completo disaccordo |
| Birth mode preference | Che tipo di parto desiderava **durante la gravidanza**? | Visual analog scale, ranging from “Sicuramente un parto naturale” (left) to “Sicuramente un parto cesareo” (right) |
| Birth mode | Come ha poi partorito? | - Con parto naturale - Con ventosa o forcipe - Con taglio cesareo |
|  | Ha dichiarato che Le è stato praticato **un taglio cesareo. Quando** è stato deciso il parto cesareo? | - Il cesareo era già stato stabilito prima del mio ricovero in ospedale. - Dopo l’inizio del travaglio. - Il taglio cesareo si è dovuto effettuare d’urgenza entro 15 minuti. |
| Indication Planned CS | Perché è stato praticato il **taglio cesareo**? Può fornire più risposte. | - Avevo problemi di salute (ad es. preeclampsia). - Taglio cesareo al parto precedente. - Peso stimato del bebè alla nascita. - Posizione del bebè (ad es. posizione podalica). - Parto gemellare (bigemino, trigemino ecc.) - Il bebè non stava bene. - È stata una mia richiesta. - Non ho capito il motivo. - Non ricordo. - Altri motivi: __________ |
| Reason elective CS | Perché **desiderava** un taglio cesareo? Può fornire più risposte. | - Paura del dolore o di complicazioni durante il parto - Paura per la sicurezza del bebè - Precedenti esperienze di parto negative - Pianificabilità della nascita - Prevenzione di lesioni gravi nella zona genitale - Altri motivi: ____________ |
| Indication unplanned CS | Ha dichiarato che Le è stato praticato un **taglio cesareo**. Perché è stato praticato il taglio cesareo? Può fornire più risposte. | - Avevo problemi di salute. - Mancata progressione del travaglio - Posizione del bebè - L'induzione del travaglio non è stata efficace. - Il bebè non stava bene. - I dolori erano troppo forti. - Ero esausta. - Non ho capito il motivo. - Non ricordo. - Altri motivi: _____________ |
| Indication instrumental vaginal birth | Perché è stato eseguito un parto con **ventosa o forcipe**? Può fornire più risposte. | - Il bebè non stava bene. - Mancata progressione del travaglio - Ero esausta. - Non ho capito il motivo. - Non ricordo. - Altri motivi:_________ |
| Duration of birth | Quante ore è durato **approssimativamente** il parto **a partire dall’inizio delle doglie regolari**? | Slider bar, ranging from “0” (left) to “48+” (right). |
| Interventions | Quali (ulteriori) misure sono state eseguite durante il parto? Può fornire più risposte. | - Induzione del travaglio. - Esami vaginali. - Monitoraggio elettronico di contrazioni e battito cardiaco (CTG). - Episiotomia (taglio chirurgico per allargare l'apertura vaginale). - Pressione sull'addome per accelerare il parto. - Apertura del sacco amniotico. - Non ricordo più. - Nessuna - Altre procedure: ______ |
| CTG | Lei ha indicato che i **suoni e le contrazioni cardiache** del Suo bebè sono stati misurati dal **CTG**. La CTG Le ha arrecato disturbo? | - Sì - No |
| Vaginal examinations | Quali affermazioni sugli **esami vaginali** alla quale è stata sottoposta sono corrette?   - È stato fatto tutto il necessario per renderli sopportabili. - Sono stata sottoposta troppo spesso a questo tipo di esami. - La mia sfera intima è stata tutelata. | Likert scale:   - completamente d’accordo - d'accordo - né d’accordo né in disaccordo - in disaccordo - in completo disaccordo |
| Indication induction of labor | Perché Le è stato **indotto il travaglio**? Può fornire più risposte. | - Il bebè non stava bene. - Diabete in gravidanza - Avevo altri problemi di salute (ad es. pressione alta, preeclampsia). - Termine superato - Il bebè è stato valutato troppo grande. - Rottura prematura delle acque e rischio di infezioni - Si sono verificati problemi con il liquido amniotico. - Il bebè non cresceva abbastanza. - Non ho capito il motivo. - Non ricordo. - Altri motivi:_________________ |
| Indication episiotomy | Perché Le è stata praticata **l’episiotomia**? Può fornire più risposte. | - Il bebè non stava bene. - Il bebè era troppo grande. - Per evitare lesioni più gravi nella zona genitale - Mancata progressione del travaglio - Non ho capito il motivo. - Non ricordo. - Altri motivi:__________ |
| Indication amniotomy | Perché Le sono state **rotte le membrane**? Può fornire più risposte. | - Accelerazione del parto - Il bebè non stava bene. - Non ho capito il motivo. - Non ricordo. - Altri motivi: __________ |
| Medication | Quali farmaci o analgesici Le sono stati somministrati? Può fornire più risposte. | - Anestesia peridurale o epidurale (riduzione del dolore attraverso il midollo spinale) - Pompa per la gestione del dolore (PCA, Happybutton) - Gas esilarante - Altri analgesici - Farmaci per l’induzione del parto - Farmaci per ridurre le contrazioni - Antibiotici - Mi è stato somministrato un farmaco, ma non so che cosa fosse. - Nessuno - Altri: _________ |
| Freedom of movement (CS) | Durante il travaglio, prima di dover essere sottoposta al taglio cesareo, ha potuto muoversi e scegliere liberamente la posizione? Può fornire più risposte. | - Sì - No, a causa dell’anestesia peridurale. - No, a causa della CTG (monitoraggio elettronico). - No, per altri motivi: __________ |
| Freedom of movement (vaginal birth) | Durante il travaglio, ha potuto muoversi e scegliere liberamente la posizione? Può fornire più risposte. | - Sì - No, a causa dell’anestesia peridurale. - No, a causa della CTG (monitoraggio elettronico). - No, per altri motivi: __________ |
| Denied procedures | Le sono state **negate** delle procedure (esami, trattamenti) durante il travaglio e il parto nonostante Lei le avesse espressamente richieste? | - No - Sì, le seguenti misure: _______ |
| Postnatal debriefing | Dopo il parto, ha avuto la possibilità di parlarne con gli specialisti coinvolti? | - Sì - Non ne ho avuto bisogno. - No, perché: ____________ |
| Postnatal debriefing helpful | Questo colloquio l’ha aiutata a chiarire o elaborare aspetti importanti? | - Sì - No |
| Maternal age | Quanti anni ha? | - 18-23 - 24-27 - 28-31 - 32-35 - 36-39 - 40+ |
| Nationality | Di che nazionalità è? | - Svizzera - Germania - Francia - Italia - Austria - Afghanistan - Africa - Asia - Belgio - Bosnia-Erzegovina - Brasile - Cina - Eritrea - Kosovo - Croazia - Macedonia - Austria - Polonia - Portogallo - Romania - Russia - Serbia - Slovacchia - Spagna - Sri Lanka - Sudamerica - Siria - Turchia - Ungheria - Stati Uniti - Regno Unito - Altro |
| Birth canton | In quale cantone è nato il Suo bebè? | - Argovia - Appenzello Esterno - Appenzello Interno - Basilea Campagna - Basilea Città - Berna - Friburgo - Ginevra - Glarona - Grigioni - Giura - Lucerna - Neuchâtel - Nidvaldo - Obvaldo - Sciaffusa - Svitto - Soletta - San Gallo - Ticino - Turgovia - Uri - Vaud - Vallese - Zugo - Zurigo |
| Marital status | Qual è il Suo stato civile? | - sposata/ in unione domestica registrata - nubile - divorziata / separata - vedova |
| Socio-economic status | Qual è all’incirca il Suo reddito familiare netto mensile? | - meno di 3'000 CHF - 3'000 – 4’999 CHF - 5'000 – 6’999 CHF - 7'000 – 8’999 CHF - 9'000 – 11’999 CHF - 12'000 – 15’000 CHF - più di 15'000 CHF |
|  | Quale scuola o formazione ha completato? | - Scuola dell’obbligo - Scuola professionale - Liceo, maturità professionale, scuola specializzata (FMS), DMS - Alta formazione tecnica e professionale - Scuola Universitaria Professionale, PH - Università, Politecnico federale (EPFL, ETH) __________ |
| Health insurance | Che assicurazione ha? | - Assicurazione di base - Semi-privata - Privata - Non so |
